# Insulin-like growth factor 1 reduces coronary atherosclerosis in pigs with familial hypercholesterolemia

**DOI:** 10.1172/jci.insight.165713

**Published:** 2023-02-22

**Authors:** Sergiy Sukhanov, Yusuke Higashi, Tadashi Yoshida, Svitlana Danchuk, Mitzi Alfortish, Traci Goodchild, Amy Scarborough, Thomas Sharp, James S. Jenkins, Daniel Garcia, Jan Ivey, Darla L. Tharp, Jeffrey Schumacher, Zach Rozenbaum, Jay K. Kolls, Douglas Bowles, David Lefer, Patrice Delafontaine

**Affiliations:** 1 Tulane University School of Medicine, New Orleans, Louisiana, USA.; 2 Cardiovascular Center of Excellence, School of Medicine, Louisiana State University, New Orleans, Louisiana, USA.; 3 Ochsner Medical Center, New Orleans, Louisiana, USA.; 4 Department of Biomedical Sciences, University of Missouri-Columbia, Missouri, USA.; 5 Faculty of Medicine, Tel-Aviv University, Tel-Aviv, Israel.

**Keywords:** Cardiology, Vascular Biology, Atherosclerosis, Growth factors, Plaque formation

## Abstract

Although murine models of coronary atherosclerotic disease have been used extensively to determine mechanisms, limited new therapeutic options have emerged. Pigs with familial hypercholesterolemia (FH pigs) develop complex coronary atheromas that are almost identical to human lesions. We reported previously that insulin-like growth factor 1 (IGF-1) reduced aortic atherosclerosis and promoted features of stable plaque in a murine model. We administered human recombinant IGF-1 or saline (control) in atherosclerotic FH pigs for 6 months. IGF-1 decreased relative coronary atheroma in vivo (intravascular ultrasound) and reduced lesion cross-sectional area (postmortem histology). IGF-1 increased plaque’s fibrous cap thickness, and reduced necrotic core, macrophage content, and cell apoptosis, consistent with promotion of a stable plaque phenotype. IGF-1 reduced circulating triglycerides, markers of systemic oxidative stress, and CXCL12 chemokine levels. We used spatial transcriptomics (ST) to identify global transcriptome changes in advanced plaque compartments and to obtain mechanistic insights into IGF-1 effects. ST analysis showed that IGF-1 suppressed *FOS/FOSB* factors and gene expression of MMP9 and CXCL14 in plaque macrophages, suggesting possible involvement of these molecules in IGF-1’s effect on atherosclerosis. Thus, IGF-1 reduced coronary plaque burden and promoted features of stable plaque in a pig model, providing support for consideration of clinical trials.

## Graphical abstract

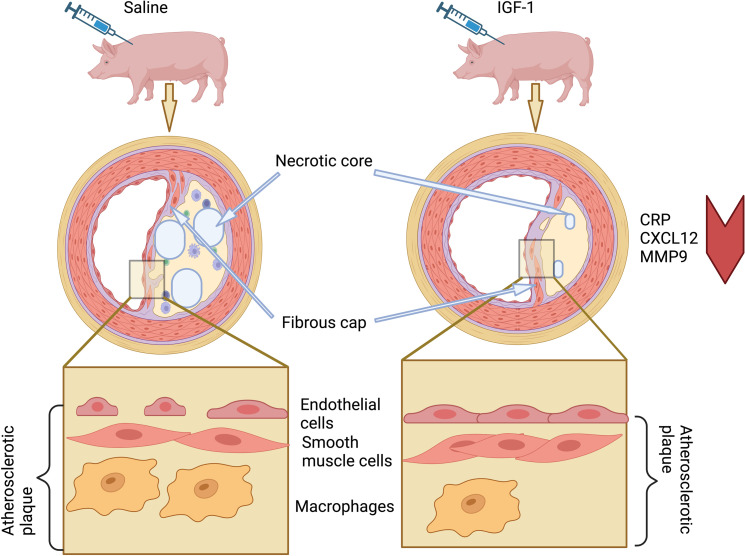

## Introduction

Despite lipid-lowering and emerging antiinflammatory agents, atherosclerosis remains the leading cause of death in both men and women in the United States and worldwide (1). Approximately every 40 seconds, someone in the United States will have a myocardial infarction, according to the 2022 heart disease statistics update (2). The estimated total cost of heart disease in the United States alone is more than $329 billion (1). Thus, interventions that reduce coronary atherosclerotic disease (CAD) would have substantial health and economic benefits.

Coronary atheroma burden is the main determinant of CAD patient outcomes (3); thus, animal models that closely resemble human coronary atherosclerotic disease are of particular interest. Although murine models have provided mechanistic insights (4–7), they have not often translated to new clinical therapies. Unlike murine models, pigs have plasma lipid profiles very close to humans (8) and develop coronary disease that is similar to humans (9). Pigs are the FDA-preferred species for testing human cardiovascular devices and the primary choice for preclinical toxicological testing of antiatherosclerotic drugs, including statins (10). Mild atherosclerotic lesions first appear in coronary arteries, and both plaque distribution and composition follow a pattern comparable to that of humans (11), with early lesions transitioning to complex plaques. Familial hypercholesterolemia is a human genetic disorder with high circulating cholesterol and low-density lipoprotein (LDL) levels, resulting in excessive atherosclerosis (12). Pigs with familial hypercholesterolemia (FH pigs) have been described by Rapacz and others (13). FH pigs harbor a point mutation in both LDL receptor alleles that reduces receptor binding, as well as allele variations in apolipoprotein B that may further contribute to the phenotype. FH swine are an excellent model for translational atherosclerosis-related research (14). Even when consuming a normal diet, FH pigs develop hypercholesterolemia and atherosclerotic lesions ranging from fatty streaks to advanced plaques, with accompanying calcification, neovascularization, hemorrhage, and rupture (11).

Insulin-like growth factor 1 (IGF-1) is an endocrine and autocrine/paracrine growth factor that has pleiotropic effects on development, metabolism, cell differentiation, and survival. There is ongoing debate on the role of IGF-1 in cardiovascular disease. Traditionally, the role of growth factors in atherosclerosis has been thought to be permissive by stimulating vascular smooth muscle cell (SMC) migration and proliferation, thereby promoting neointima formation (15). Some cross-sectional and prospective studies suggest a positive association between IGF-1 and atherogenesis (16), but others have found that low IGF-1 is a predictor of ischemic heart disease and mortality, consistent with the potential antiatherosclerotic and plaque stabilization effects of IGF-1 (17). Methodological constraints could explain these contradictions because measurement of total IGF-1 levels represents only a crude estimate of the biologically active IGF-1. IGF-1 levels negatively correlate with body weight (BW) and age, and this additionally confounds examination of the role of IGF-1 in cardiovascular disease.

We have shown that IGF-1 is an antiatherogenic factor in mice (18). Systemic IGF-1 infusion reduced aortic root plaque area by approximately 30% in apolipoprotein E–knockout (Apoe^–/–^) mice (19). This effect is similar to that of statins in the same mouse model (e.g., high-dose rosuvastatin, ref. 20), and statins greatly reduce acute coronary events in patients with atherosclerosis (21). Consistent with our results, another group reported that long arginine-3 IGF-1 increased plaque SMCs and cap thickness and reduced the rate of intraplaque hemorrhage, indicating that IGF-1 promotes plaque stabilization (17). These findings are in line with most clinical studies (22, 23) but not all (24), which have suggested that lower circulating IGF-1 levels and higher levels of IGF-1 binding protein 3 are associated with increased risk of atherosclerotic disease. Validation of our murine studies in a large animal model is critical to consider use of IGF-1 to treat atherosclerosis in humans. Antiatherosclerotic effects of novel drugs are routinely tested in pigs (including statins, ref. 25); however, to our knowledge there are no reports directly testing IGF-1 effects on atherogenesis in a large animal model. Furthermore, since epidemiological studies have reported a positive association between circulating IGF-1 levels and various primary cancers, such as breast, colorectal, and prostate cancer (26), it is critical to examine potential carcinogenic effects of long-term IGF-1 administration in a large animal model.

Here, we document IGF-1 effects on coronary atherosclerosis in FH pigs, using recombinant human IGF-1 at a dose that is FDA approved for long-term treatment of growth failure in children with severe primary IGF-1 deficiency. Furthermore, we use spatial transcriptomics (ST), an innovative groundbreaking technology that quantifies changes in the whole transcriptome within a morphological context (27), to detect spatially and differentially expressed genes targeted by IGF-1. Our results provide mechanistic insights into IGF-1–induced effects on atherosclerosis and to our knowledge represent the first report on the use of ST to analyze atherosclerotic tissue from animals or humans.

## Results

### Phenotype of FH pigs.

**Figure 1:**
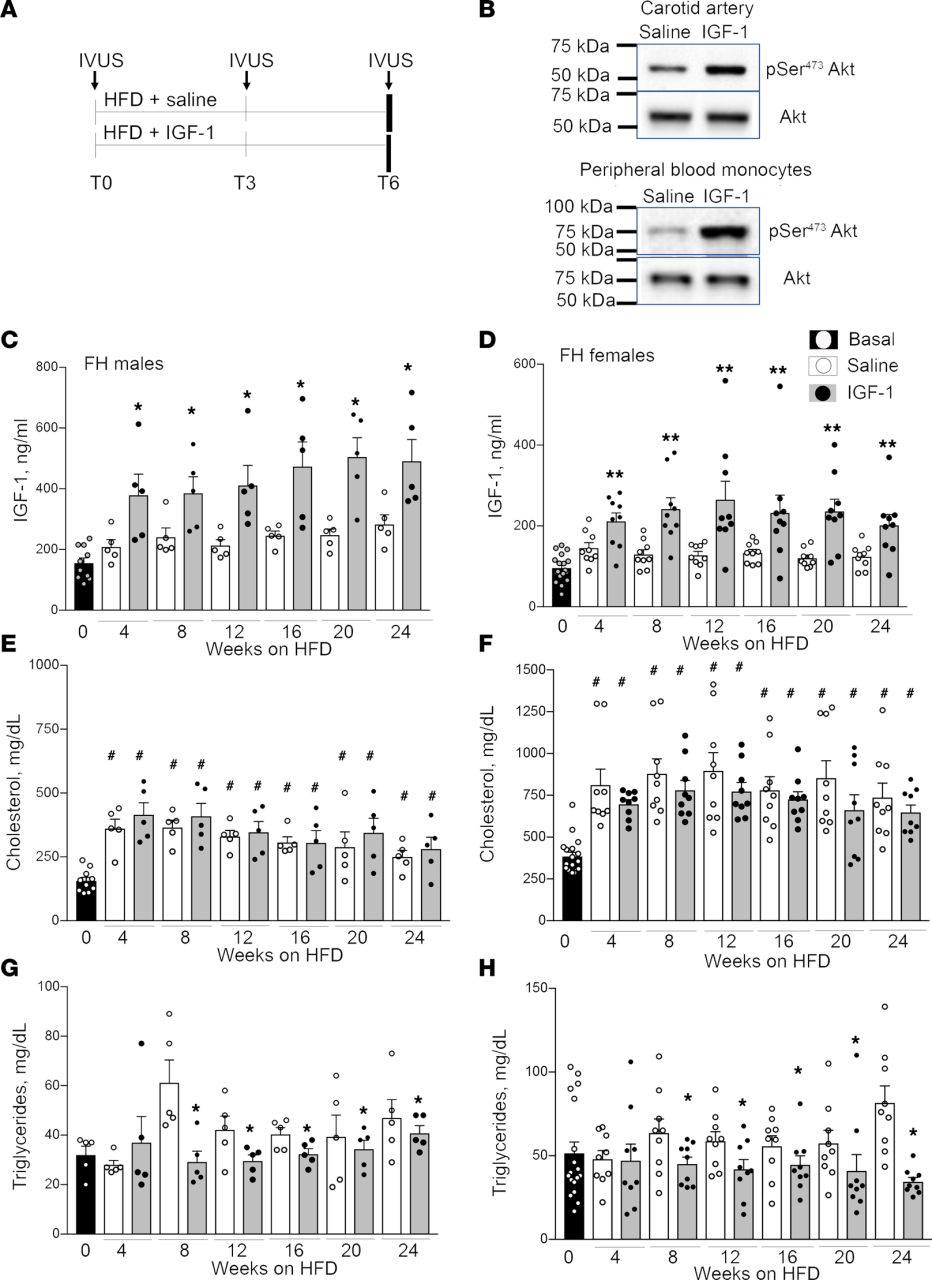
Phenotype of FH pigs. (**A**) Experimental design. FH pigs were injected daily with 50 μg/kg human recombinant IGF-1 or saline (control) (males, *n* = 5/group; females, *n* = 9/group) and fed with high-fat diet (HFD) for 6 months. Coronary atherosclerosis was quantified by intravascular ultrasound (IVUS) before injections (T0), after 3 months (T3), and after 6 months (T6, at sacrificing). (**B**) IGF-1 stimulated specific downstream signaling in porcine carotid artery and in peripheral blood mononuclear cells (PBMC). IGF-1 (or saline) was injected into pig, carotids and blood were collected 4 hours following injections, and Akt phosphorylation was quantified by immunoblotting. (**C**–**H**) Blood was collected at basal level (T0) and each month during injections (total 7 time points). Total plasma IGF-1 level was quantified by ELISA in FH males (**C**) and females (**D**). Cholesterol (**E** and **F**) and triglyceride (**G** and **H**) levels in IGF-1– and saline-injected male (left) and female (right) FH pigs. Males: *n* = 10 for basal, and *n* = 5/group for each time point. Females: *n* = 18 for basal, and *n* = 9/group for each time point. *P < 0.05, **P < 0.01, vs. saline based on *t* test, ^#^*P* < 0.05 vs. basal level based on 3-way ANOVA.

We used 14-month-old FH noncastrated males (*n* = 5/group) and gilt (never been used for breeding) females (*n* = 9/group) and administered recombinant human IGF-1 (rhIGF-1), 50 μg/kg/d, twice per day, or saline for 6 months (Figure 1A). Of note, the IGF-1 dose is within the range of the FDA-approved dosage for long-term treatment of growth failure in children with primary IGF-1 deficiency (28), and the amino acid sequence structure of porcine IGF-1 is identical to human IGF-1 (29). Safety, pharmacokinetics, and efficacy of IGF-1 are reported for patients (28, 30, 31). To verify that IGF-1 administration into FH pigs stimulates specific downstream signaling in porcine vasculature and blood cells, we injected recombinant human rhIGF-1 (or saline, control) into pigs and isolated carotid arteries and peripheral blood mononuclear cells (PBMCs) after 4 hours. Akt phosphorylation was increased by almost 4-fold (*P* < 0.001) in both vascular tissue and PBMC sin IGF-1–injected pigs versus control (Figure 1B), indicating that IGF-1 promotes specific downstream signaling. IGF-1 levels at T0 were higher in males versus females (Figure 1, C and D, *P* < 0.005). IGF-1–injected pigs had significantly higher IGF-1 levels compared with control pigs at all tested time points. The average increase in IGF-1 group versus saline in males was 88.0% ± 19.4% and 83.3% ± 9.7% in females.

Male and female animals had a similar initial BW on average, and both IGF-1– and saline-injected pigs gained BW steadily throughout the study, with no difference found between saline and IGF-1 groups (Supplemental Figure 1; supplemental material available online with this article; https://doi.org/10.1172/jci.insight.165713DS1). FH males had a larger BW increase compared with FH females (M, 69.2% ± 2.9% increase at T6 versus T0; F, 45.0% ± 5.2% increase, *P* < 0.01).

Blood pressure and heart rate were measured in sedated pigs at each IVUS procedure. We found no difference in systolic/diastolic blood pressure and heart rate between sexes and between saline and IGF-1 groups (Supplemental Table 1). There was no time-dependent change in blood pressure or in heart rate.

Blood tests were performed monthly. We found no statistically significant difference in complete metabolic profiles and complete blood count (CBC) with differential between sexes and saline versus IGF-1 group (Supplemental Tables 2 and 3). However, we found that females had a striking 2.3-fold higher cholesterol levels at T0 compared with males (*P* < 0.001, Figure 1, E and F). HFD feeding caused a significant and sustained elevation of total cholesterol levels in both sexes. IGF-1 did not change total cholesterol in both males and females. FH females had significantly higher triglyceride levels compared with males at T0 (*P* < 0.005, Figure 1, G and H), and HFD feeding did not change triglycerides. IGF-1 reduced triglycerides in FH pigs (3-way ANOVA, *P* < 0.005, Figure 1, G and H).

### Necropsy/histopathology findings.

IGF-1 levels have been reported to be associated with an increased risk of cancer (26); thus, we performed autopsies and collected all major organs for histopathological analysis. No gross abnormalities were found either in saline-injected or IGF-1–injected FH pigs, and no tissue was considered carcinogenic by a certified pathologist. All female pigs had multiple coalescing yellow to tan plaques on the intimal surface of the aorta and large visible lipid deposits in the right coronary artery (RCA), left anterior descending artery (LAD), and circumflex artery. Two female pigs (saline, 1; IGF-1, 1) had evidence of a myocardial infarction. Abdominal fat deposits, hepatic lipidosis/fatty liver, and fatty lymph nodes were found in several saline- and IGF-1–injected females. Two female pigs in the saline group had pleural adhesions and moderate splenic enlargement, suggesting inflammation. Sections of the lung, liver, kidney, spleen, lymph node, stomach, small and large intestines, urinary bladder, ovary, uterus, pancreas, salivary gland, skeletal muscle, thyroid, and aorta from each animal were examined. No significant microscopic lesion including inflammatory or neoplastic process was noted in all animals (not shown) except atherosclerosis in the aorta and in coronary arteries.

### IGF-1 decreases coronary atherosclerosis.

IVUS was performed in the RCA and LAD at T0, T3, and T6 time points (Figure 1A). We found no significant difference in vessel volume, lumen volume, and plaque + media volume between RCA and LAD and between sexes at T0 (Table 1). There was a time-dependent increase of the vessel volume, presumably due to normal animal growth and to vascular remodeling concomitant with intimal thickening. In fact, the lumen volume time dependently decreased in both saline- and IGF-1–injected pigs (*P* < 0.001 for both RCA and LAD), consistent with intimal thickening. IGF-1–injected pigs had a larger time-dependent increase in RCA and LAD artery volume compared with controls (*P* < 0.05), suggesting vascular hypertrophy. Coronary arteries in IGF-1–injected FH females had larger lumen volume at T3 and T6 (*P* < 0.005) compared with control.

**Figure 2:**
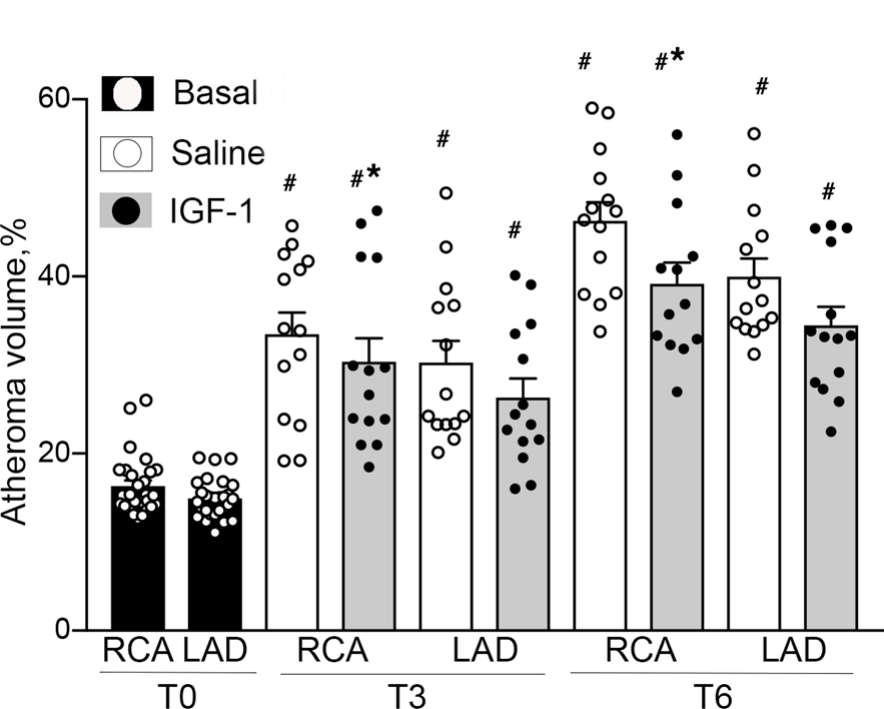
IGF-1 reduces coronary atheroma volume. FH pigs (both sexes) were injected with IGF-1 or saline (control) (males, 5/group; females, 9/group). The coronary atheroma volume was quantified in RCAs and LADs by serial IVUS before injections (T0), after 3 months (T3), and after 6 months (T6). Relative atheroma volume (%) was defined as plaque + media volume divided per the vessel (external elastic membrane, EEM) volume × 100%. Since sex does not influence IGF-1’s effect on relative atheroma volume, atheroma measurements of both sexes were combined and shown. *n* = 28/group for T0, and *n* = 14 per RCA or LAD per group for T3 and T6. *P < 0.05 vs. saline, and ^#^*P* < 0.05 vs. T0 based on 2-way ANOVA.

Pigs had approximately 16% of relative atheroma volume at T0. IGF-1– and saline-injected pigs had a time-dependent increase in absolute plaque + media volume and in relative atheroma volume (*P* < 0.001 for RCA and LAD). FH females had a significantly larger time-dependent increase in RCA and LAD relative atheroma at T3 (females, 36.6% ± 2.6%, vs. males, 23.3% ± 0.2%) and at T6 (females, 47% ± 4% vs. males, 35.9% ± 1.9%), indicating the presence of a strong sex effect. IGF-1 did not significantly change the absolute plaque + media volume in RCA and LAD. We found no interaction between IGF-1 effect on relative atheroma and sex (3-way ANOVA). As sex does not influence IGF-1’s potential effect on relative atheroma volume, we combined measurements of both sexes and performed a 2-way analysis (treatment vs. time) using Bonferroni’s correction for repeated measurements. IGF-1 time dependently decreased relative atheroma volume in RCA (*P* < 0.05 vs. saline) and in LAD (*P* = 0.054, Figure 2). The IGF-1–induced increase in vessel lumen and reduction in relative atheroma indicate that IGF-1 reduces coronary atherosclerosis.

**Figure 3:**
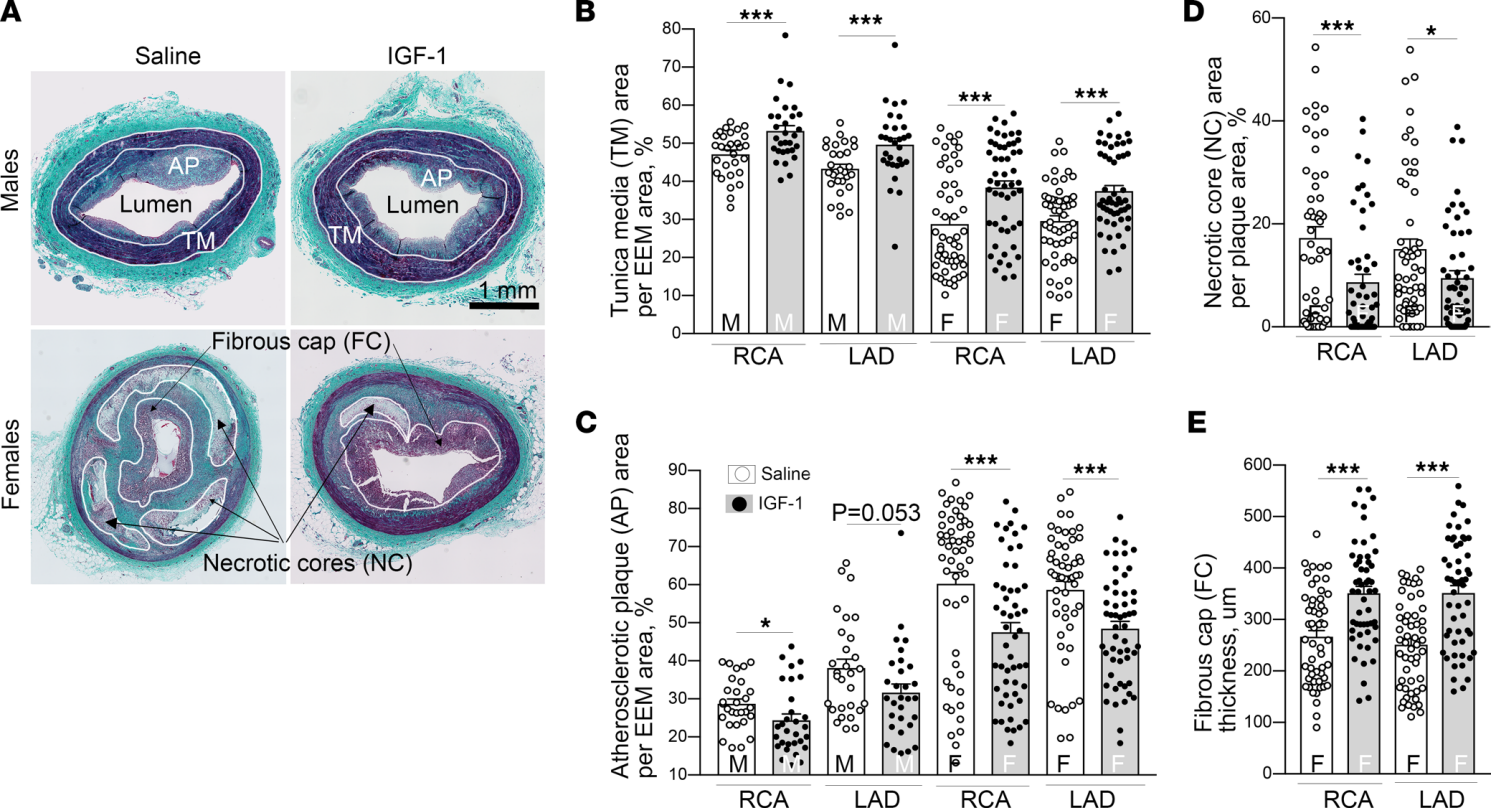
IGF-1 reduces coronary atherosclerosis and promotes features of stable atherosclerotic plaque. IGF-1 increased vascular media (**A** and **B**), reduced atherosclerotic plaque cross-sectional area (CSA) (**C**), decreased necrotic core (**D**), and elevated thickness of fibrous cap (**E**). RCA and LAD were isolated from IGF-1– and saline-injected FH pigs and further cut onto 6 sequential fragments for embedding in paraffin. Trichrome-stained cross sections were obtained from each fragment and used for morphological analysis. *n* = 30 per RCA or LAD per group for males and *n* = 54 for females. (**A**) Representative images of RCA sections obtained from FH males and females. Tunica media (TM), atherosclerotic plaque (AP), fibrous cap (FC), and necrotic cores (NCs) were manually outlined to quantify TM and AP CSA, and results were normalized per EEM area. The thickness of FC was calculated as the mean length of 5 arbitrary lines distributed across the cap area. *P < 0.05, ***P < 0.005 vs. saline based on unpaired 2-tailed *t* test.

Trichrome-stained RCA and LAD cross sections were used for histological analysis. FH females developed larger and more complex plaques in coronaries compared with males, in agreement with IVUS results (Figure 3). Plaques in males contained diffuse homogeneous collagen material and lipid droplets and had neither necrotic cores nor fibrous caps. We classified plaques in FH males as type III pre-atheroma in accordance with histological classification of human atherosclerotic lesions (32). Coronary plaques in FH females were significantly larger (RCA: 2-fold increase in CSA versus males, LAD: 1.5-fold increase, *P* < 0.05, data for saline group); they contained dense fibrous caps, large acellular/necrotic cores, and multiple cholesterol clefts. We observed the presence of strong calcification (Alizarin Red staining, data not shown) and neovascularization (IHC with CD31, endothelial cell marker, data not shown) in coronary plaques in FH females but not in males. Plaques in FH females were classified as advanced type V fibroatheroma (32).

FH males had a thicker tunica media compared with females in both IGF-1– and saline-injected groups (*P* < 0.05 in each case, Figure 3B). IGF-1 significantly increased medial CSA (males, RCA: 13% increase vs. control; LAD: 12% increase; females, RCA: 34% increase; LAD: 26% increase), consistent with vascular hypertrophy (Figure 3B). IGF-1 did not change total vessel CSA (outlined by external elastic membrane boundary, data not shown). IGF-1 reduced relative atherosclerotic plaque area in males (RCA, 15.2% ± 4.5% decrease; LAD, 16.6% ± 7.1% decrease compared with control) and in females (RCA, 21.5% ± 2.7% decrease; LAD, 17.4% ± 2.1% decrease compared with control) (Figure 3C), consistent with IVUS data. Plaques in IGF-1–injected females were more cellular and contained reduced necrotic cores compared with controls (RCA: 50.1% ± 1.6% decrease; LAD: 47.8% ± 1.4% reduction, Figure 3D). IGF-1 significantly increased the thickness of fibrous caps in coronary plaques in female pigs (Figure 3E). Our results indicate that IGF-1 induces vascular hypertrophy, reduces coronary atherosclerotic burden, and promotes features of plaque stability.

### IGF-1 reduces macrophage-like cells and upregulates endothelial-like cells in coronary plaque.

We reported previously that IGF-1 increases plaque SMCs (33), downregulates macrophages (MFs), and elevates levels of circulating endothelial cell (EC) progenitors (19) in HFD-fed Apoe^–/–^ mice. SMCs and MFs share cell markers in the atherosclerotic plaques (34), and plaque ECs undergo a change in phenotype toward a mesenchymal cell type (35). Such phenotype switching complicates marker-based cell identification. To validate the IHC protocol, serial RCA sections were stained with a set of cell marker antibodies and immunopositivity pattern was compared. We found that each of 4 SMC marker antibodies stained virtually identical cell populations in the plaque, and a similar conclusion was made for 3 tested MF and 3 EC marker antibodies (Supplemental Figure 2). These data show that IHC with antibody for a single cell marker identified plaque cells expressing multiple markers, increasing confidence in identifying specific plaque cells. We also verified that cells immunopositive for macrophage scavenger receptor A (MSR), an MF marker, were immunonegative for α–smooth muscle actin (α-SMA), an SMC marker, and vice versa (Supplemental Figure 2A), showing that these antibodies have no cross-reactivity.

**Figure 4:**
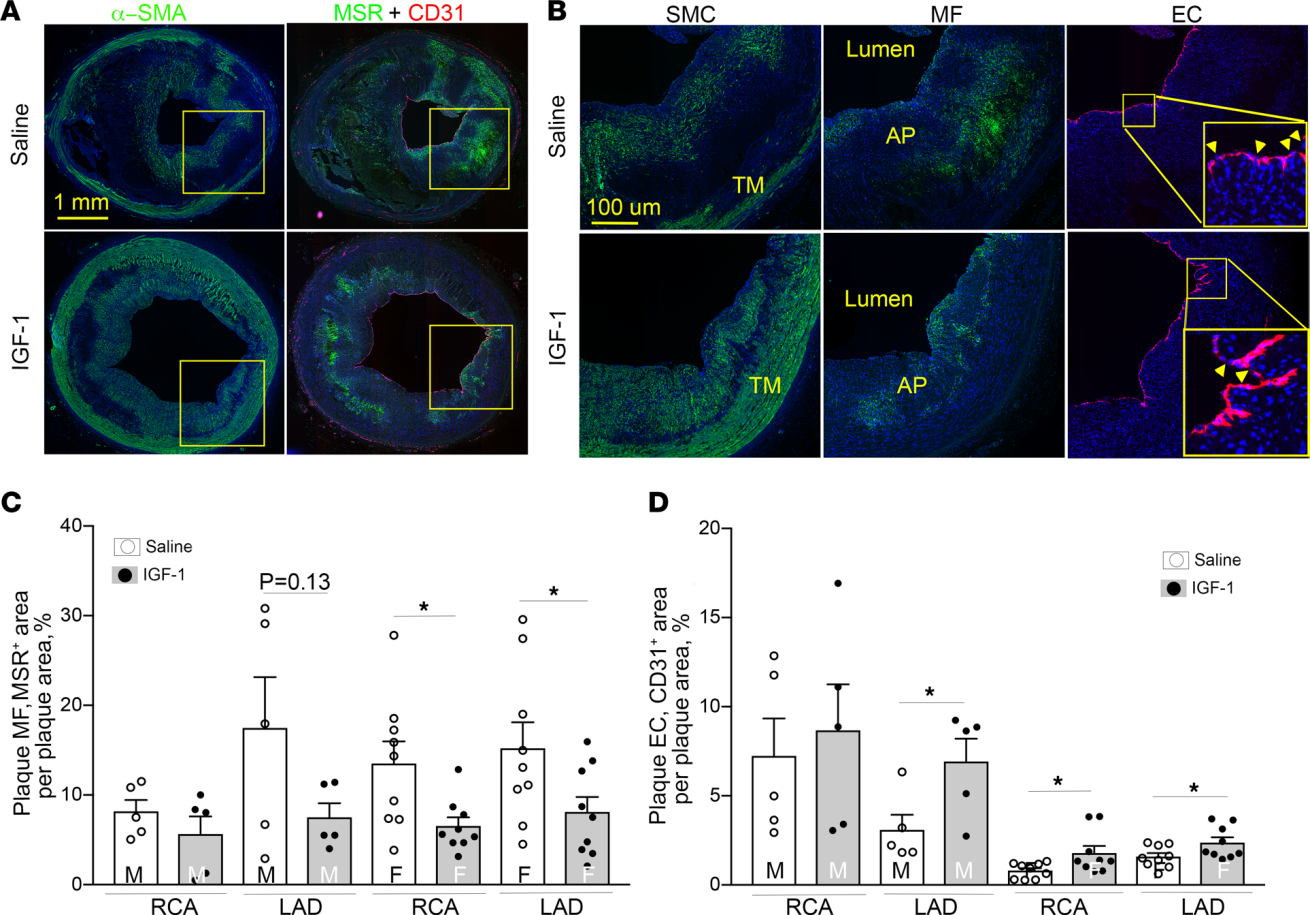
IGF-1 suppresses MF-like cells and upregulates EC-like cells in coronary plaques. Serial RCA and LAD sections were immunostained with α–smooth muscle actin (α-SMA), macrophage scavenger receptor A (MSR), and CD31 antibody to identify SMC-like, MF-like, and EC-like cells, respectively. The primary antibody signal was amplified by biotin/streptavidin or tyramide systems conjugated to Alexa Fluor 488 (for α-SMA and MSR) or Alexa Fluor 594 (CD31). (**A**) Representative images of RCA sections obtained from IGF-1– or saline-injected FH females. Yellow square outlines plaque area magnified in **B**. (**B**) SMC, MF, and EC marker–immunopositive cells. Yellow arrows in insert indicate breaks in endothelial layer. (**C** and **D**) Quantitative data. *n* = 5 per RCA or LAD per group for males and *n* = 9 for females. *P < 0.05 vs. saline based on unpaired 2-tailed *t* test.

We used α-SMA, MSR, and CD31 antibodies to quantify SMC-like, MF-like, and EC-like cells, respectively, by IHC. SMC-like cells were abundant in the vascular media, and a mixture of SMC- and MF-like cells was found in the plaque fibrous cap (Figure 4 and Supplemental Figure 2A). In addition, MF-like cells were present in the area surrounding the plaque necrotic core and colocalized with cholesterol clefts in lipid cores. IGF-1–injected pigs had a slight increase in plaque SMC-like cells (*P* = NS) (Supplemental Figure 3) and a dramatic 2-fold reduction in plaque MF-like cells in females (*P* < 0.05 for RCA and LAD) (Figure 4). IGF-1 increased EC marker–positive area and this effect reached significance for RCA and LAD in females and LAD in males (Figure 4). Notably, EC marker–positive area was markedly larger in males compared with females (Figure 4A). We noted discontinuous CD31^+^ staining in endothelium layers of both IGF-1– and saline-injected pigs, suggesting either CD31 downregulation, or focal loss of EC, suggesting reduced endothelial integrity. To further obtain a surrogate index of endothelium layer integrity, we normalized CD31^+^ area per lumen perimeter. IGF-1 significantly increased CD31^+^ area/lumen perimeter ratio in the RCA and LAD in the female group (pixels^2^/pixels, RCA: IGF-1, 5.04 ± 0.84 vs. saline, 2.76 ± 0.64, *P* < 0.05; LAD: IGF-1, 10.31 ± 1.53 vs. saline, 5.52 ± 0.51, *P* < 0.01), suggesting that IGF-1 reduced the number of CD31^+^ endothelium layer breaks.

Systemic IGF-1 administration increased expression of pro–α 1(I) collagen in aortic lysates (36), and SMC-specific IGF-1 overexpression increased collagen fibrillogenesis in the atherosclerotic plaque in Apoe^–/–^ mice (37). We found that IGF-1–injected pigs had a trend toward increased collagen levels in the vascular media and in coronary plaques (~10% increase, *P* = NS) (Supplemental Figure 3, B and C). Thus, IGF-1 changed the cellular composition of porcine coronary plaques. Atherosclerotic lesions in IGF-1–injected pigs had decreased levels of MF-like cells and increased endothelial-like cells compared with controls.

### IGF-1 decreases cell apoptosis, reduces systemic oxidative stress, and suppresses inflammation.

**Figure 5:**
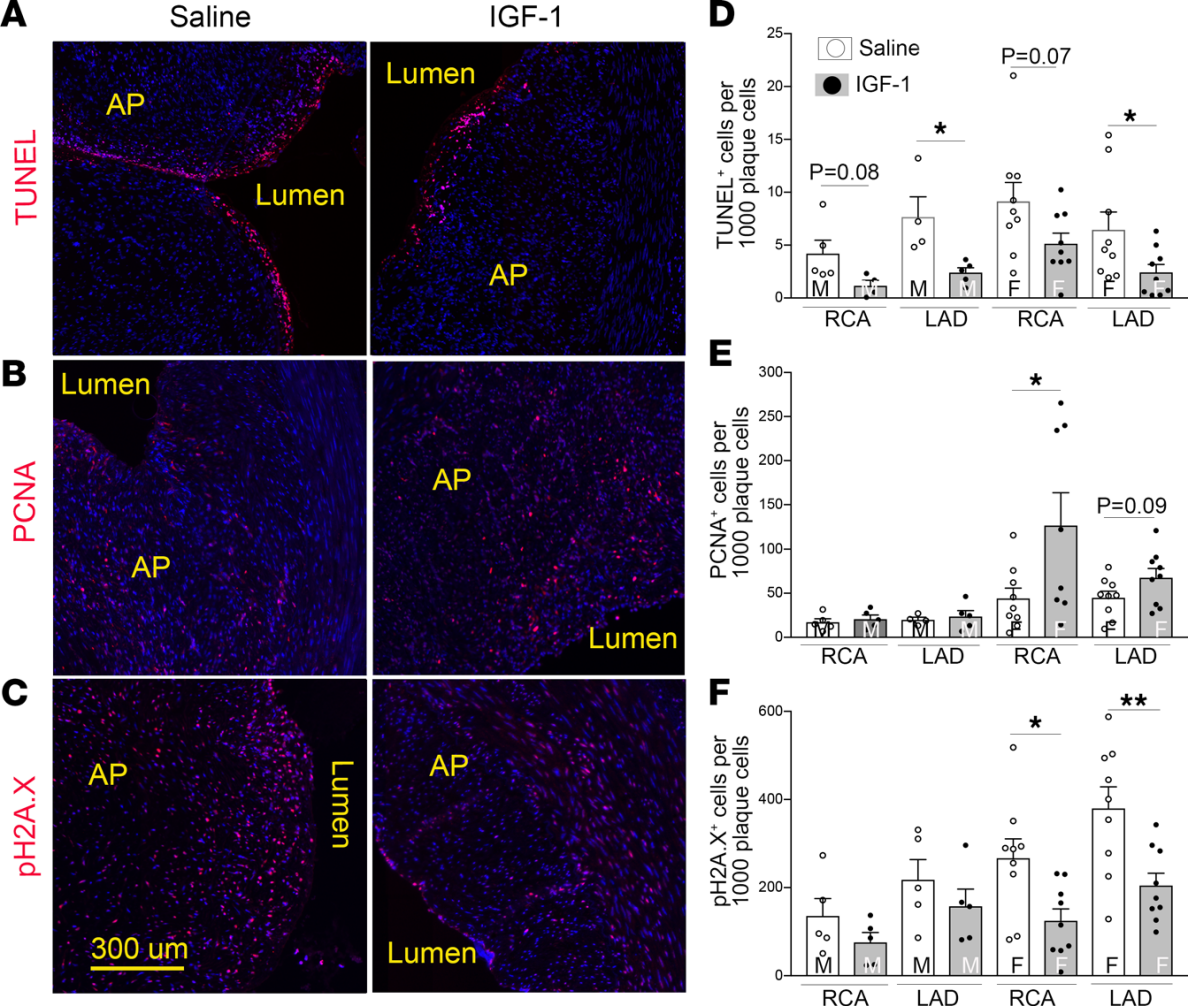
IGF-1 suppresses plaque cell apoptosis, promotes cell proliferation, and decreases DNA damage. Cell apoptosis was quantified by TUNEL assay, and cell proliferation and DNA damage were quantified by immunostaining with PCNA antibody and pH2A.X antibody, respectively. (**A**–**C**) Representative images of RCA sections obtained from IGF-1– or saline-injected FH females. (**D**–**F**) Quantitative data. *n* = 5 per RCA or LAD per group for males and *n* = 9 for females. *P < 0.05, **P < 0.01 vs. saline based on unpaired 2-tailed *t* test.

IGF-1 is a mitogen and prosurvival molecule (38) and exerted antiapoptotic, antioxidant, and antiinflammatory effects in Apoe^–/–^ mice (19, 33). Apoptotic cells in porcine coronary plaques were localized on the luminal border, in the fibrous cap (Figure 5A), and around necrotic cores. IGF-1–injected pigs had an almost 3-fold decrease in cell apoptosis in the male group (*P* < 0.05 for LAD) and an approximately 2-fold reduction in apoptosis rate in females (Figure 5D). Proliferating cell nuclear antigen (PCNA) is a cell proliferation marker. Atherosclerotic plaques in the female group had increased PCNA immunopositivity compared with males. IGF-1 upregulated PCNA levels in coronary plaques in females (*P* < 0.05 in RCA) and did not change the PCNA signal in the male group (Figure 5, B and E).

**Figure 6:**
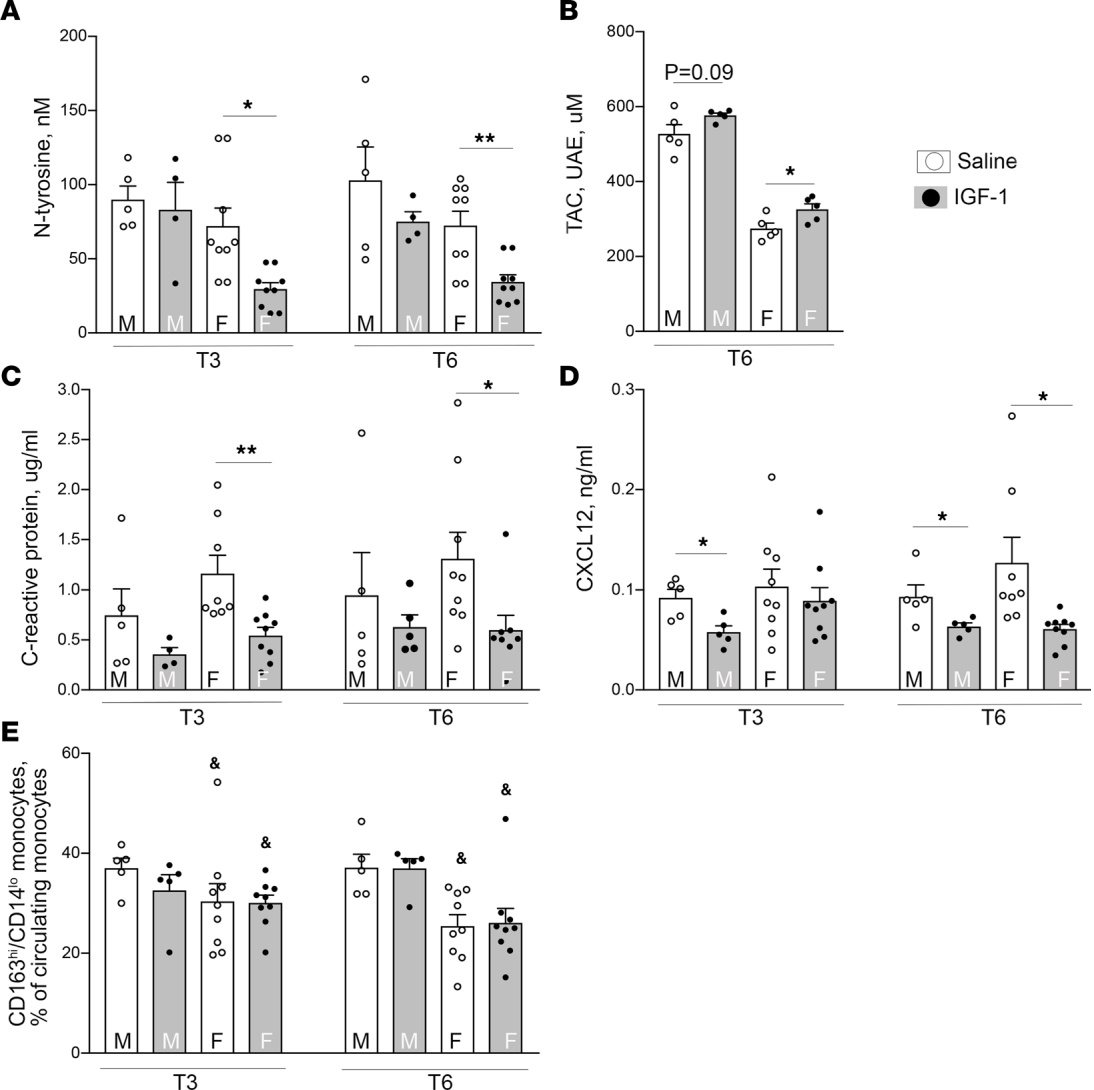
IGF-1 downregulates markers of systemic oxidative stress and decreases C-reactive protein and chemokine CXCL12. Markers of systemic oxidative stress (**A** and **B**), C-reactive protein (CRP) (**C**), and chemokine CXCL12 (**D**). IGF-1 did not alter circulating monocyte subsets (**E**). Circulating N-tyrosine, CXCL12, and CRP levels were quantified by ELISA and TAC, by using colorimetric assay. TAC assay results shown in urinary acid (standard) equivalents (UAE). (**E**) Whole blood was mixed with a cocktail of antibodies against CD163-PE, CD14–Alexa Fluor 488, and porcine CD172a and subsequently with streptavidin-APC/Cy7. CD172a-positive leukocytes were size-gated and further differentiated into subsets based on CD163 and CD14 expression levels using FACS. *n* = 5 per time point per group for males and *n* = 9 for females for N-tyrosine, CRP, CXCL12, and monocyte assay. *n* = 5/males and females for TAC assay. *P < 0.05 and **P < 0.01 vs. saline, ^&^*P* < 0.05 vs. males based on 3-way ANOVA.

Oxidative stress is a major characteristic of hypercholesterolemia-induced atherosclerosis (39). Oxidative DNA damage promotes cell apoptosis and contributes to formation of unstable plaques. Histone H2A.X phosphorylation is a highly specific molecular marker to quantify DNA damage (40). We found that 15%–35% of plaque cells in coronaries contained detectable levels of phosphorylated S139-histone H2A.X (pH2A.X) (Figure 5, C and F), and higher pH2A.X levels correlated with larger atherosclerotic burden seen in females. IGF-1 significantly decreased the number of pH2A.X^+^ cells in plaques in the female group. Circulating levels of N-tyrosine and total plasma antioxidant capacity (TAC) were measured as indices of systemic oxidative stress (Figure 6). IGF-1 decreased plasma N-tyrosine levels in females at both T3 and T6 time points (56% and 47% decrease, respectively, vs. saline, *P* < 0.05) (Figure 6A). FH female pigs had a 2-fold reduction in TAC compared with males at T6. IGF-1 upregulated TAC in males and females (Figure 6B), though the increase in males did not reach statistical significance. Taken together, the N-tyrosine and TAC data indicate that IGF-1 suppressed systemic oxidative stress.

CRP is an acute marker of inflammatory responses, and circulating levels of CRP correlate with progression of CAD (41). Human CRP transgene expression causes accelerated aortic atherosclerosis in Apoe^–/–^ mice (42). FH females had higher circulating CRP levels compared with males. IGF-1 significantly decreased CRP levels of both sexes at T3 and T6 (Figure 6C), suggesting a reduction of inflammatory responses. Macrophage-specific IGF-1 reduces chemokine CXCL12 levels, and this effect is associated with decreased atherosclerotic burden in Apoe^–/–^ mice (43). IGF-1 significantly reduced circulating CXCL12 in FH males at T3 and T6 time points and in the female group at T6 (Figure 6D).

The frequency of monocyte subsets has been linked to severity of atherosclerosis in patients with stable CAD (44). We measured 2 subsets of circulating monocytes, defined by surface expression levels of CD163 and CD14. Monocyte subpopulations were assessed by flow cytometry. CD172a^+^ myeloid cells were size-gated to monocytes, which were further assessed for CD163 and CD14 expression levels, classifying cells as CD163^hi^CD14^lo^ monocytes and CD163^lo^CD14^hi^ monocytes. The population size of CD163^hi^CD14^lo^ monocytes was larger in male animals than in female animals (*P* < 0.001) (Figure 6). No significant effects were induced by IGF-1 administration.

### IGF-1 changes the global transcriptomic profile of coronary plaques.

To obtain further insights into the effects of IGF-1 on coronary atherosclerosis, we performed an exploratory analysis of advanced plaques from FH females, using the newly developed technology spatial transcriptomics. ST provides an unbiased picture of entire transcriptome changes within a spatial context (27). Prior to running ST analyses, we confirmed the quality of RNA preparations. Total RNA was extracted from plaque-containing RCA cryosections (*n* = 4), and RNA integrity number (RIN) (45) was quantified using an Agilent Bioanalyzer. RIN was greater than 7, which is considered suitable for ST analysis according to the manufacturer’s (Visium, 10x Genomics) recommendations. RCA cryosections from IGF-1– and saline-injected FH females (*n* = 2/group) were processed, and ST quality controls (Supplemental Table 4) were consistent with a successful experiment. Correlation of ST gene expression with protein expression (measured by IHC) was verified for selected gene/protein combinations, including IGF-1 binding protein 7 (IGFBP7) and α-SMA (data not shown). Furthermore, changes in gene expression of MMP9, IGFBP7, and FOS measured by ST were validated by real-time PCR using aliquots of mRNA isolated from tissue sections (data not shown).

**Figure 7:**
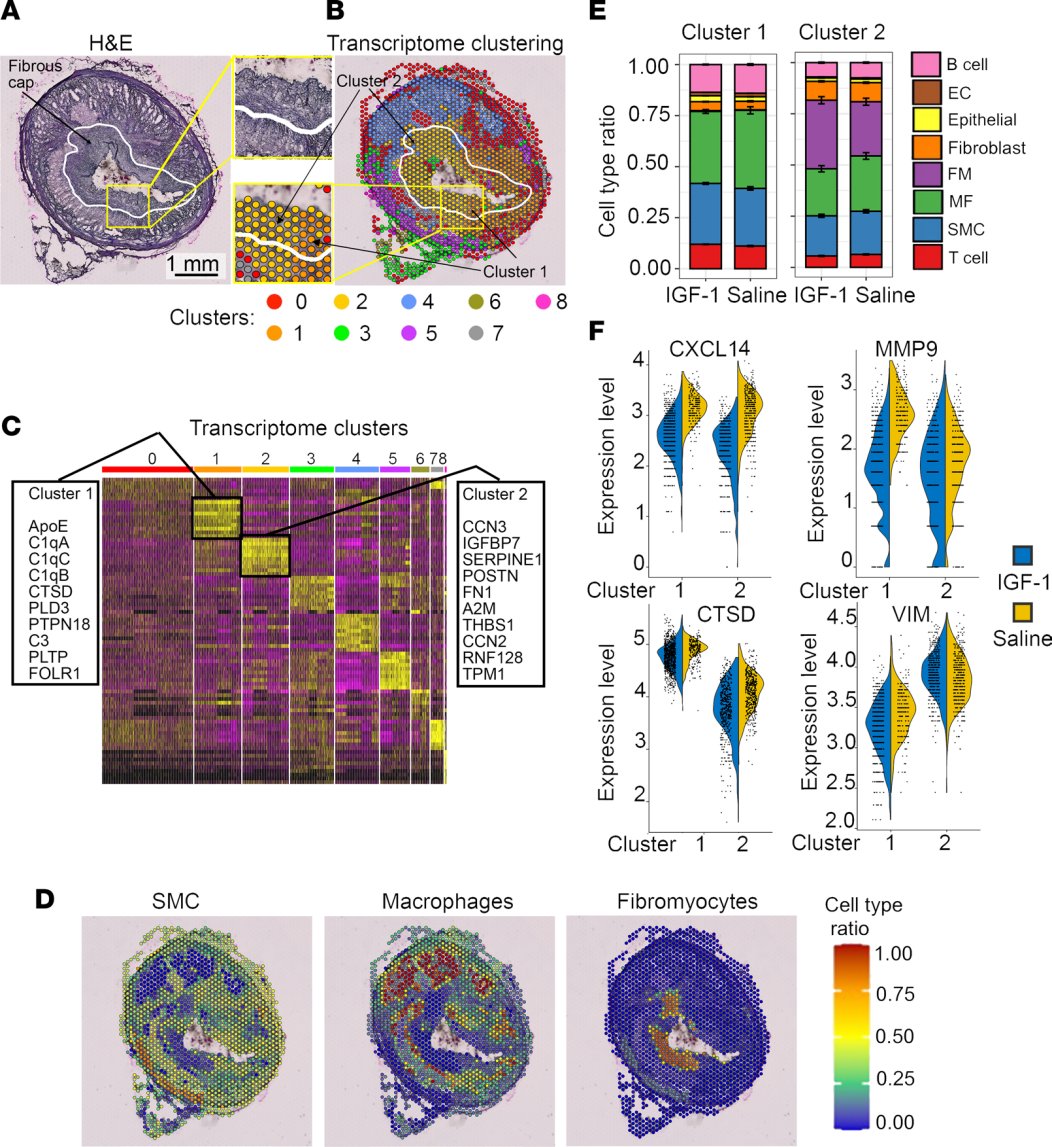
IGF-1 alters global transcriptomic profile of coronary plaque. The RCA cryosections were obtained from IGF-1–injected (*n* = 2) and saline-injected (*n* = 2) FH females, and spatially resolved global transcriptome was assessed by ST. ST spots in IGF-1 and saline specimens were grouped into 9 clusters (numbered 0–8) based on their transcriptome, and the heatmap with top 10 upregulated genes/cluster was generated. (**A**) H&E-stained image with FC outline (white curve). (**B**) Representative transcriptome clustering. Yellow squares in **A** and **B** outline FC fragment shown magnified between panels. Cluster 1 and 2 were identified within a histologically homogeneous FC area. (**C**) Heatmap. (**D**) Cell type ratio was calculated for each ST spot to identify spots enriched by SMCs, MFs, or fibromyocytes (FMs). (**E**) Cell type ratio for IGF-1 versus saline specimens for cluster 1 and 2. (**F**) Violin plots show expression levels of CXCL14, MMP9, cathepsin D (CTSD), and vimentin (VIM) comparing IGF-1 versus saline specimens in cluster 1 and 2. IGF-1 downregulated CXCL14, MMP9, VIM, and CTSD within cluster 1 and decreased expression of CXCL14, MMP9, and CTSD within cluster 2 compared with saline.

We performed unsupervised clustering of all ST spots in IGF-1 and saline specimens by using a manufacturer-suggested algorithm with R toolkit Seurat (46). ST spots were grouped into 9 clusters (numbered 0–8) based on their transcriptome (Figure 7B). We identified the top 10 genes overexpressed in each cluster (versus all other clusters) to obtain the heatmap (Figure 7C). In parallel, plaque FC, necrotic/lipid core, tunica media, and tunica adventitia were outlined using H&E images, and transcriptome clusters and histological annotations were compared side by side. FC contained mainly (94.4%) transcriptome cluster 1 and 2, and tunica adventitia cluster 0 and 3 (Figure 7B and Supplemental Figure 4A).

The mixed cell deconvolution algorithms use single-cell RNA-sequencing (scRNA-Seq) data as a reference to characterize cellular heterogeneity in a spatial context (47). However, to our knowledge, there are no scRNA-Seq data available for pig atherosclerotic specimens. Therefore, we used scRNA-Seq data obtained for human atherosclerotic RCA (Gene Expression Omnibus GSE131778) (48) as a reference to assess the cellular composition in our porcine ST data set. Using a deconvolution algorithm (48), we calculated the cell type ratio for each ST spot to identify spots enriched in SMCs (SMC-high), MFs (MF-high), or fibroblast-like cells, termed fibromyocytes (FM-high) (Figure 7D). Of note, we observed good agreement between localization of ST-defined SMC- and MF-high spots and IHC-detected immunopositivity pattern obtained for cell markers. The cell type ratio shows that transcriptomic cluster 4 contained around 80% MF-high spots, cluster 5 had more than 70% SMC-high spots, and virtually all FM-high spots (>95%) were assigned to cluster 2 (Supplemental Figure 4B).

Table 2 shows the top up- and downregulated genes in IGF-1 versus saline specimens identified in all ST spots, and Table 3 contains a list of DEGs in SMC-, MF-, and FM-high spots. IGF-1 dramatically (>10-fold reduction vs. saline) downregulated FOS and FOSB proto-oncogenes in all ST spots and in SMC-high spots. IGF-1 downregulated expression of the cytoskeletal molecule desmin in all spots and upregulated ribosomal protein S27 (Table 2), a component of the translational machinery (49). Activation of translation is in line with known growth-stimulating IGF-1 action (38). MMP9 gene expression level was reduced by IGF-1 in all ST spots and in MF-high spots. This is consistent with our recent report that MF-specific IGF-1 overexpression downregulates MMP9 in peritoneal MFs and decreases aortic atherosclerosis in Apoe^–/–^ mice (43). Cytokine CXCL14 was the top IGF-1–downregulated gene in MF-high spots (Table 3). CXCL14 was upregulated in MF-derived foam cells, and CXCL14 peptide-induced immunotherapy suppresses atherosclerosis in Apoe^–/–^ mice (50), suggesting a proatherogenic role of CXCL14.

We observed a clear boundary between transcriptome cluster 1 and 2 within FC histological area (Figure 7, A and B), showing the capability of ST analysis to identify plaque areas with different gene expression patterns that are not histologically distinguishable. To take advantage of this likely unique ST feature and considering the importance of the FC for overall stability of atheroma (51), we focused our subsequent analysis on cluster 1 and 2. First, we found discrete differences in the cellular composition of cluster 1 and 2. More T cells, B cells, and MFs were in cluster 1, whereas fibroblast levels were lower (Supplemental Figure 4B). The top upregulated genes in cluster 1 (versus other clusters) included components of the complement system (C3, C1qA, C1qC, and C1qB) and cathepsin D (*CTSD*; Figure 7C). The top genes in cluster 2 included CCN2 and CCN3 (CCN molecules are involved in wound healing and fibrosis, ref. 52) and fibronectin 1, which is known to play a vital role in tissue repair (53). A comparison of DEGs between cluster 1 and 2 showed that upregulated *CTSD* was the top-ranked DEG in cluster 1 versus 2 (~2.1-fold higher expression in cluster 1 vs. 2; adjusted *P* value = 1.71 × 10^–220^). CTSD is a proapoptotic molecule and collagen-degrading protease that is highly expressed in MFs (54). Higher cathepsin activity is associated with unstable plaque (54). Ingenuity Pathway Analysis (IPA; QIAGEN) predicted that necrosis and apoptosis pathways are upregulated in cluster 1 versus 2 (data not shown), consistent with increased expression of CTSD. Importantly, cluster 1 is the thinnest part of the FC. Taken together, these data suggest that cluster 1 is a tissue-degrading and less fibrotic compartment of the FC compared with cluster 2. We speculate that cluster 1 represents a plaque site with potentially increased vulnerability and propensity to erode or rupture.

We also compared cell type ratio and DEGs in cluster 1 and 2 in IGF-1 versus saline specimens. IGF-1 slightly decreased MFs in both cluster 1 and 2, and IGF-1 upregulated FMs in cluster 2 (Figure 7E). SMC transition into FMs prevents FC thinning (48), suggesting that higher FM levels in the FC are beneficial during the disease process. IGF-1 reduced CTSD expression in both clusters, whereas vimentin expression (VIM) was decreased only in cluster 1. The top-ranked DEGs in comparing IGF-1 versus saline groups in cluster 1 and 2 included downregulated chemokine CXCL14 and MMP9 (Figure 7F). Thus, use of ST combined with deconvolution algorithms provided, to our knowledge for the first time, a profile of the spatial transcriptome of advanced atherosclerotic plaque and changes induced by IGF-1.

## Discussion

To our knowledge, the current study is the first to test the effects of IGF-1 given long term in a large animal model of atherosclerosis. We found that IGF-1 induced vascular hypertrophy, reduced coronary atheroma volume, and decreased plaque CSA over 6 months and that there was no evidence of IGF-1–induced tumorigenesis over this period. There was evidence of significant sex-specific differences in atherosclerosis development. Females had higher cholesterol and triglyceride levels and an advanced plaque phenotype. IGF-1 increased FC thickness, and reduced necrotic core size, macrophage content, and cell apoptosis, changes consistent with promotion of a more stable plaque phenotype. IGF-1 also reduced circulating triglycerides and markers of systemic oxidative stress. ST analysis of plaques from female FH pigs, combined with a mixed cell deconvolution algorithm, allowed us to spatially profile gene expression in advanced coronary plaques and to identify DEGs in IGF-1–treated animals. We detected 9 specific gene clusters and found that the plaque FC was composed almost exclusively of cluster 1 and 2, showing the potentially unique capability of ST to identify plaque subcompartments that are not histologically distinguishable. IGF-1 induced marked decreases in FOS/FOSB transcription factor and in MMP9 and CXCL14 gene expression in plaque macrophages, suggesting involvement of these molecules in IGF-1’s antiatherogenic effects.

Although multiple studies have shown that IGF-1 exerts antiatherosclerotic effects in murine models (17, 18), the role of IGF-1 in the development of human atherosclerotic disease is unclear (22, 23). Both IGF-1 administration and gain-of-function and loss-of-function approaches in mice have provided mechanistic insights, but consideration of IGF-1 for treatment of cardiovascular disease in humans mandates demonstration of IGF-1’s efficacy and safety in a large animal model that is physiologically closer to humans.

We administered IGF-1 at a dose approved for use in children with primary IGF-1 deficiency (28). FH males were noncastrated to fully evaluate potential sex-dependent effects. Coronary plaque location, size, and frequency (as assessed by coronary angiography, data not shown) were similar to ones reported for humans (55). Coronary atheroma cellular composition, presence of large necrotic/lipid cores, neovascularization, and calcification in FH females closely resembled the phenotype of advanced fibroatheromas reported for patients with CAD (56). These data demonstrate that FH pigs are a valuable model to study CAD and test antiatherosclerotic drugs.

The number of preclinical studies comparing plaque development between the sexes is extremely limited relative to the vast literature exploring atherosclerosis mechanisms (57). FH females had reduced circulating IGF-1 levels and higher plasma cholesterol and triglyceride levels compared with males. HFD feeding caused a substantial and sustained elevation of total cholesterol levels in both sexes. The causative role of high cholesterol in promoting atherogenesis has been well established using multiple animal models, including miniature pigs (58, 59) and genetically modified mice and rabbits (60, 61). High cholesterol and triglycerides are classical risk factors for human CAD (62). We hypothesize that high lipid levels in FH females were a major cause of the increased atherosclerosis in this group, though it is possible that lower basal IGF-1 levels in female pigs also increased susceptibility to atherosclerosis development. Of note, although IGF-1 had no effect on total cholesterol (Figure 1, E and F), it reduced triglycerides (Figure 1, G and H). Our study is, to our knowledge, the first to report higher cholesterol levels and atherosclerotic burden in FH females compared with males. Further studies will be required to establish mechanisms mediating these sex-specific differences.

We demonstrated that IGF-1 exerted atheroprotective effects on pre-atheroma (in males) and advanced fibroatheromas (in females). Accumulating evidence suggests that endothelial dysfunction is an early marker for atherosclerosis, and damage of coronary endothelium has been shown to constitute an independent predictor of cardiovascular events (63). The barrier function of the endothelium is impaired in atherosclerosis, leading to uncontrolled leukocyte extravasation and vascular leakage (64). Intriguingly, we found that the IGF-1–induced antiatherosclerotic effect in FH pigs was associated with reduced EC damage. Indeed, we found multiple breaks in the EC layer in porcine plaque, indicating loss of endothelial integrity. IGF-1–injected pigs had a reduced number of EC layer breaks in coronary plaques, suggesting improved EC function. This result is in line with our recent report showing that EC-specific IGF-1 receptor deficiency downregulated endothelial intercellular junction proteins, elevated endothelial permeability, and enhanced atherosclerotic burden in Apoe^–/–^ mice (65). Increased EC lining in plaques in IGF-1–treated pigs is potentially due to elevated EC proliferation or increased recruitment of circulating EC progenitors to the plaque area. IGF-1 has been shown to promote EC proliferation (66) and increase levels of EC progenitors in Apoe^–/–^ mice (19).

We found that IGF-1 exerted antioxidant and antiapoptotic effects in FH pigs, which may have contributed to attenuation of atherosclerosis progression, leading to smaller necrotic core size and a more stable plaque phenotype. Indeed, IGF-1 reduced plasma N-tyrosine levels, increased TAC, and concomitantly reduced the number of plaque TUNEL^+^ and pH2A.X^+^ cells. Of note, these findings are consistent with the ability of IGF-1 to upregulate expression of the antioxidant enzyme glutathione peroxidase in cultured ECs (67) and to downregulate 12/15-lipoxygenase levels in murine plaques (68), suggesting that these mechanisms may be relevant to the effects of IGF-1 in the FH pig model. Furthermore, elevated SMC apoptosis has been associated with low IGF-1 expression in human advanced plaques (69), and IGF-1 receptor activation inhibits oxidized lipid-induced apoptosis in SMCs through the PI3K/Akt signaling pathway (70), indicating its involvement in IGF-1–induced suppression of apoptosis.

Single-cell transcriptomic analysis of human atherosclerotic plaques has been reported (71), but critical information linking changes in cell-specific transcriptome with plaque morphology is missing. ST captures all polyadenylated transcripts and provides an unbiased picture of entire transcriptome changes within a spatial context (27). Thus, ST encodes positional information onto transcripts before sequencing, allowing visualization of expression of virtually any gene of interest within the original tissue section (27). There are ST-generated spatial atlases of disease-free organs and diseased tissues (cancer, Alzheimer’s disease, amyotrophic lateral sclerosis, and rheumatoid arthritis) (27). However, there are no published reports to our knowledge using ST with atherosclerotic tissue obtained from animal models or humans. We used FH pig coronary plaque specimens to optimize the ST technology, and quality controls obtained at the end of experiment correlated well with the manufacturer’s recommendations. We found good agreement between transcriptomic profiles and protein expression data detected by IHC. ST revealed distinct clusters of gene expression within histologically homogeneous parts of the plaque, e.g., the FC, which contained almost exclusively cluster 1 and 2 with a clear boundary between these clusters. Cluster 1 had a gene expression pattern, cellular content, and a morphological feature (reduced thickness) suggesting elevated sensitivity of this site to plaque rupture. Further studies will be required to determine the clinical relevance of this finding.

ST combined with the cell deconvolution algorithm identified genes differentially expressed in response to IGF-1 in spots enriched in SMCs, MFs, and FMs. ST identified FOS and FOSB genes as molecules with the highest IGF-1–induced reduction in expression among all ST spots and SMC-high spots. A recent study showed that c-FOS (protein encoded by the *FOS* gene) is involved in oxidized lipid-induced formation of SMC-derived foam cells. SMC-specific c-FOS deficiency prevents formation of foam cells in vitro and suppresses atherosclerosis in HFD-fed Apoe^–/–^ mice (72), demonstrating a fundamental proatherogenic role of FOS. *FOS* and *FOSB* encode leucine zipper proteins that dimerize with JUN proteins, thereby forming the transcription factor complex activator protein-1 (AP-1). In vitro and in vivo studies have implicated AP-1 as a critical common inflammatory transcription factor mediating progression of atherogenesis (73) and have suggested that AP-1 inhibition is a promising strategy to treat atherosclerosis (74). AP-1 was reported to mediate oxidized lipid-induced CXCL14 upregulation in MF-derived foam cells (50). Here we report that IGF-1–induced FOS/FOSB downregulation correlated with CXCL14 gene expression decrease in MF-high spots. CXCL14 is known to bind and activate another proatherogenic cytokine, CXCL12 (75). We reported recently that MF-specific IGF-1 overexpression decreases CXCL12 plaque and circulating levels and promotes atherosclerosis in mice (43). Of note, in the current study, we found that IGF-1 markedly decreased circulating CXCL12. Taken together, these results suggest that downregulation of the AP-1 complex and suppression of the CXCL14/CXCL12 axis are potential mechanisms contributing to IGF-1–induced antiatherogenic effects.

Most acute coronary events are related to rupture or erosion of atherosclerotic plaques that are not hemodynamically significant (76). Thus, plaque stability is a critical determinant of clinical events. Stable plaques are characterized by increased collagen, reduced apoptosis, thicker FC, smaller necrotic cores, and decreased number of inflammatory cells (56). We have shown that coronary plaques in IGF-1–injected FH pigs had reduced necrotic cores, increased FC thickness, decreased levels of MF-like cells, and reduced cell apoptosis, and these changes are consistent with promotion of a stable plaque phenotype. We also found that IGF-1 decreased the number of MF-high spots, reduced gene expression of CXCL14 chemokine, and downregulated MMP9 gene expression specifically in cluster 1, a potentially more vulnerable component of the FC. Taken together, these results suggest that IGF-1 has substantial plaque-stabilizing properties.

In summary, we report here that IGF-1 administered over 6 months decreased coronary atherosclerosis and promoted features of stable atheroma in FH pigs, without evidence of a tumorigenic effect. IGF-1 stimulated an array of potentially antiatherogenic mechanisms, including suppression of oxidative stress, systemic inflammatory response, and cell apoptosis. Furthermore, IGF-1 decreased EC damage in coronary plaques. ST technology combined with cell deconvolution analysis identified an area of the FC with potentially more propensity for erosion or rupture. Furthermore, ST revealed that IGF-1 induced major changes in the plaque transcriptome. IGF-1 dramatically suppressed gene expression of FOS/FOSB transcription factors and of CXCL14 chemokine and MMP9 in plaques, and these data suggest involvement of these molecules in mediating IGF-1 effects. Our results provide mechanistic insights into IGF-1–induced effects on atherosclerosis, are a critical step in taking IGF-1 to human studies, and to our knowledge represent the first report on the use of ST to analyze atherosclerotic tissue from animals or humans.

## Methods

Extended methods are available in the Supplemental Methods.

### Animals.

The *Rapacz* familial hypercholesterolemic swine (*Sus scrofa*) (FH pigs) were received from the Swine Research and Teaching Center at University of Wisconsin. We administered recombinant human IGF-1 (INCRELEX, IPSEN) 50 μg/kg, s.c. every 12 hours, twice per day) to FH pigs for 6 months. The control group received an equal volume of saline (Figure 1A). We used a total of 28 FH pigs for experiments (males, *n* = 5 per group; females, *n* = 9 per group). All pigs received an HFD starting the day after T0. We performed serial IVUS at T0 and at T3 and T6 to assess coronary atherosclerotic burden (Figure 1A). FH pigs were sacrificed after T6 IVUS. We followed guidelines of American Veterinary Medical Association for the euthanasia of animals (2020 edition) (77).

### IVUS analysis.

IVUS was performed using the IVUS imaging system (Volcano Corporation) and a 20 MHz 3.5 F Visions PV 0.035 Digital IVUS Catheter (Volcano Corporation). For the current study 20 mm of IVUS pullback segment distal to the ostia of the RCA and the LAD was selected. The area circumscribed by the outer border of the echolucent tunica media and the luminal border was manually traced on each 1 mm IVUS frame within selected fragment. The following indices of vessel morphology were assessed: 1) lumen volume (mm³) = lumen area (the area bounded by the luminal border) × length of fragment; 2) vessel volume (mm³) = EEM area × length of fragment; 3) plaque + media volume (mm³) = vessel volume – lumen volume; 4) relative atheroma volume (%) = plaque plus media volume divided by the vessel volume × 100%.

### Atherosclerotic burden and plaque composition analysis.

The entire RCA and LAD were collected, and the proximal 30 mm fragment of RCA and LAD was further cut onto six 5 mm fragments for embedding in paraffin. Histological blocks were sequentially labeled 1–6; serial 6 μm cross sections were cut from each block and stained with Gomori’s trichrome stain (Polysciences Inc). Sections from each block were used for morphological analysis. EEM, internal elastic membrane, and luminal border were manually outlined in CellSens Dimension 1.18 software (Olympus Corp), and corresponding CSAs were measured by 2 independent researchers, 1 of them under a blinded protocol. The cellular content of coronary plaques was assessed by IHC with serial sections obtained from the middle (no. 3) coronary fragment. Cell marker–specific antibodies for α-SMA (MilliporeSigma, CBL171, clone ASM1), MSR (TransGenia Inc, KT022, clone SRA-E5), and CD31 (Abcam, 134168, clone EP3095) were used for IHC to identify SMCs, MFs, and ECs, respectively. Proliferating cells and cells with DNA damage were quantified by IHC with antibody against PCNA (MilliporeSigma, MAB424R, clone PC10) and pH2A.X (Abcam, ab2893), respectively. Cell apoptosis was quantified with In Situ Cell Death Detection Kit, TMR red (MilliporeSigma, 12156792910), as per manufacturer’s instructions.

### Blood biochemistry.

Fasting blood samples were collected from the jugular vein at baseline and monthly. Blood was collected in 0.1 mol/L citrate-containing EDTA. Fresh whole blood was submitted to Antech Diagnostics for CBC with differential and biochemistry measurements (Superchem w/CBC, SA020). Plasma IGF-1 levels were quantified by human IGF-I Quantikine ELISA Kit (R&D Systems, DG100B) and CRP levels by porcine C-reactive protein/CRP DuoSet ELISA (R&D Systems, DY2648). Quantification of plasma N-tyrosine and TAC assay were performed with OxiSelect Nitrotyrosine ELISA Kit (STA-305) and TAC assay kit (STA-360) (both from Cell Biolabs Inc).

### ST.

ST uses spotted arrays of specialized mRNA-capturing probes containing a spatial barcode unique to that spot. When a cryosection is attached to the slide, the capture probes bind mRNA from the adjacent point in the tissue. After mRNA extraction, a cDNA library is generated and sequenced. ST was conducted using the Visium Spatial Gene Expression System (10x Genomics) in accordance with manufacturer’s instructions. RCA cryosections (IGF-1, *n* = 2, saline, *n* = 2) were H&E-stained, and mRNA was extracted to ST array, followed by cDNA synthesis, library construction, and sequencing with Illumina NextSeq 1000 system (800 million paired-end reads). Pig reference genome was created from *Sus scrofa* genomic sequence (Sscrofa 11.1) and Ensembl annotation, and reads were aligned and counted by Space Ranger (10x Genomics; ver 2.0.0). ST data were deposited to Gene Expression Omnibus (GSE220218). ST quality was verified by the percentage of valid barcodes, Q30 bases in barcodes, valid unique molecular identifiers, and reads mapped to the genome (Supplemental Table 4). All the downstream analyses were performed using R toolkit Seurat (46) and IPA software (QIAGEN). The mixed cell deconvolution was performed in accordance with the algorithm developed by Wirka et al. using scRNA-Seq data obtained for human atherosclerotic RCAs (GSE131778) (48) as a reference. Differential gene expression analysis was performed using Seurat’s FindMarkers function, which identifies DEGs based on the nonparametric Wilcoxon rank-sum test. *P* values were adjusted by Bonferroni’s correction. Error bars indicate SEM.

### Statistics.

Statistical comparisons for histology data were performed by unpaired 2-tailed *t* test. IVUS results and blood biochemistry data were analyzed using a 3-way repeated measures ANOVA taking treatments (saline vs. IGF-1 administration), time (periodical, repeated measurements), and animal’s sex (female vs. male) as variables. Grubb’s *z* test was used to identify outliers. Data sets were first assessed for residual distribution using D’Agostino-Pearson omnibus normality test and for equal variances using Levene’s test for equality of variances. Differences in outcomes were determined by ANOVA and Bonferroni’s multiple comparisons test, Kruskal-Wallis test, or Mann-Whitney *U* test, accordingly with the normality of residual distribution. For all comparisons, *P* < 0.05 was considered statistically significant. Adjusted *P* value (78) was used for statistical comparison of genomic data sets obtained with ST. Data were analyzed using Microsoft Excel and Prism v.6.0 (GraphPad Software). Data presented in figures are individual data points (circles) and mean ± SEM (bars). Artwork was generated in GraphPad Prism and Adobe Photoshop 15.0.

### Study approval.

All animal experiments were performed in accordance with the *Guide for the Care and Use of Laboratory Animals* (National Academies Press, 2011), the Public Health Service Policy on the Humane Care and Use of Laboratory Animals, and the Animal Welfare Act. Institutional Animal Care and Use Committee approvals were obtained from the University of Missouri-Columbia and Louisiana State University Health Sciences Center New Orleans, New Orleans, Louisiana, USA, before initiation of experimental studies.

## Author contributions

SS designed the study, performed histological assays, performed IVUS analysis, ran ST, and wrote the manuscript; YH designed the study, performed blood biochemistry and monocyte subset assessment, and contributed to writing the manuscript; SD and MA carried out ELISAs; TY and JKK handled ST data, and ran bioinformatics; TG, AS, TS, JSJ, DG, JI, DLT, and DB performed IVUS surgery; JS carried out necropsy; ZR assisted with IVUS analysis; and DB, DL, and PD designed the study and contributed to writing the manuscript.

## Supplementary Material

Supplemental data

## Figures and Tables

**Table 1:**
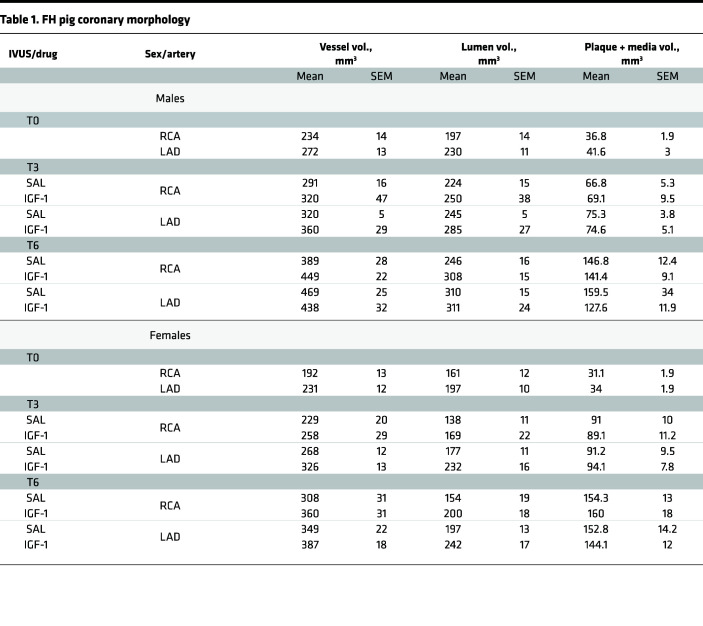
FH pig coronary morphology

**Table 2:**
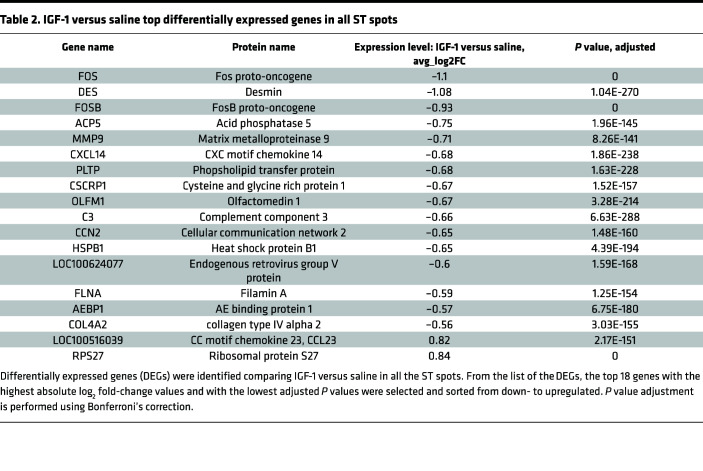
IGF-1 versus saline top differentially expressed genes in all ST spots

**Table 3:**
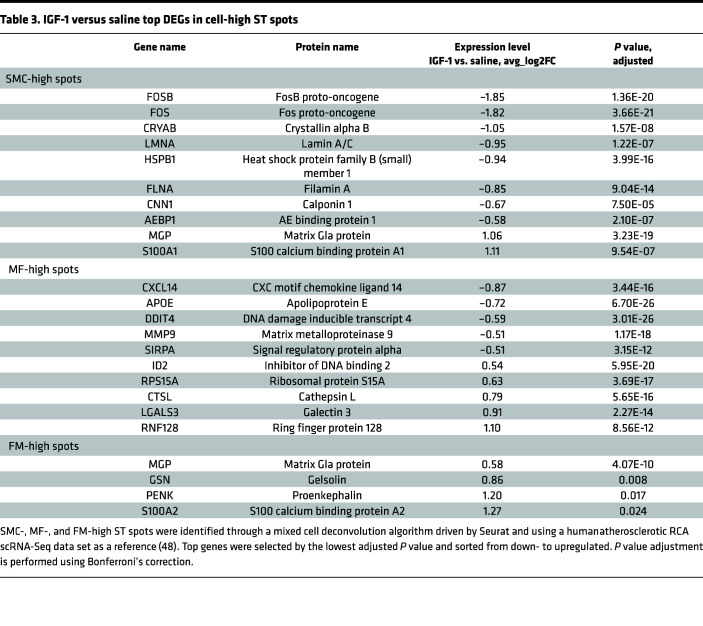
IGF-1 versus saline top differentially expressed genes in all ST spots

## Version 1 01/05/2023

In-Press Preview

## Version 2 02/22/2023

Electronic publication

## References

[1] Mozaffarian D (2016). Executive summary: heart disease and stroke statistics--2016 update: a report from the American Heart Association. Circulation.

[2] Tsao CW (2022). Heart disease and stroke statistics-2022 update: a report from the American Heart Association. Circulation.

[3] Arbab-Zadeh A (2018). Coronary atheroma burden is the main determinant of patient outcome: but how much detail is needed?. Circ Cardiovasc Imaging.

[4] Sui W (2019). Bladder drug mirabegron exacerbates atherosclerosis through activation of brown fat-mediated lipolysis. Proc Natl Acad Sci U S A.

[5] Zhang C (2010). Angiotensin-converting enzyme 2 attenuates atherosclerotic lesions by targeting vascular cells. Proc Natl Acad Sci U S A.

[6] Dong M (2013). Cold exposure promotes atherosclerotic plaque growth and instability via UCP1-dependent lipolysis. Cell Metab.

[7] Cao R (2003). Angiogenic synergism, vascular stability and improvement of hind-limb ischemia by a combination of PDGF-BB and FGF-2. Nat Med.

[8] Kaabia Z (2018). Plasma lipidomic analysis reveals strong similarities between lipid fingerprints in human, hamster and mouse compared to other animal species. Sci Rep.

[9] Suzuki Y (2011). The representative porcine model for human cardiovascular disease. J Biomed Biotechnol.

[10] Dixon JL (2002). Increased atherosclerosis in diabetic dyslipidemic swine: protection by atorvastatin involves decreased VLDL triglycerides but minimal effects on the lipoprotein profile. J Lipid Res.

[11] Prescott MF (1991). Development of complex atherosclerotic lesions in pigs with inherited hyper-LDL cholesterolemia bearing mutant alleles for apolipoprotein B. Am J Pathol.

[12] Schwartz CJ (1993). A modern view of atherogenesis. Am J Cardiol.

[13] Hasler-Rapacz JO (1994). Familial and diet-induced hypercholesterolemia in swine. Lipid, ApoB, and ApoA-I concentrations and distributions in plasma and lipoprotein subfractions. Arterioscler Thromb.

[14] Hamamdzic D, Wilensky RL (2013). Porcine models of accelerated coronary atherosclerosis: role of diabetes mellitus and hypercholesterolemia. J Diabetes Res.

[15] Ross R (1987). Growth factors in the pathogenesis of atherosclerosis. Acta Med Scand Suppl.

[16] Ruotolo G (2000). Serum insulin-like growth factor-I level is independently associated with coronary artery disease progression in young male survivors of myocardial infarction: beneficial effects of bezafibrate treatment. J Am Coll Cardiol.

[17] Von der Thusen JH (2011). IGF-1 has plaque-stabilizing effects in atherosclerosis by altering vascular smooth muscle cell phenotype. Am J Pathol.

[18] Higashi Y (2019). IGF-1 and cardiovascular disease. Growth Horm IGF Res.

[19] Sukhanov S (2007). IGF-1 reduces inflammatory responses, suppresses oxidative stress, and decreases atherosclerosis progression in ApoE-deficient mice. Arterioscler Thromb Vasc Biol.

[20] Schroeter MR (2009). Rosuvastatin reduces atherosclerotic lesions and promotes progenitor cell mobilisation and recruitment in apolipoprotein E knockout mice. Atherosclerosis.

[21] Raber L (2015). Effect of high-intensity statin therapy on atherosclerosis in non-infarct-related coronary arteries (IBIS-4): a serial intravascular ultrasonography study. Eur Heart J.

[22] Juul A (2002). Low serum insulin-like growth factor I is associated with increased risk of ischemic heart disease: a population-based case-control study. Circulation.

[23] Schuler-Luttmann S (2000). Insulin-like growth factor-binding protein-3 is associated with the presence and extent of coronary arteriosclerosis. Arterioscler Thromb Vasc Biol.

[24] Kawachi S (2005). Circulating insulin-like growth factor-1 and insulin-like growth factor binding protein-3 are associated with early carotid atherosclerosis. Arterioscler Thromb Vasc Biol.

[25] Lee DL (2003). Increased endothelin-induced Ca2+ signaling, tyrosine phosphorylation, and coronary artery disease in diabetic dyslipidemic Swine are prevented by atorvastatin. J Pharmacol Exp Ther.

[26] Shanmugalingam T (2016). Is there a role for IGF-1 in the development of second primary cancers?. Cancer Med.

[27] Rao A (2021). Exploring tissue architecture using spatial transcriptomics. Nature.

[28] Fintini D (2009). Profile of mecasermin for the long-term treatment of growth failure in children and adolescents with severe primary IGF-1 deficiency. Ther Clin Risk Manag.

[29] Tavakkol A (1988). Porcine insulin-like growth factor-I (pIGF-I): complementary deoxyribonucleic acid cloning and uterine expression of messenger ribonucleic acid encoding evolutionarily conserved IGF-I peptides. Mol Endocrinol.

[30] Keating GM (2008). Mecasermin. BioDrugs.

[31] Khwaja OS (2014). Safety, pharmacokinetics, and preliminary assessment of efficacy of mecasermin (recombinant human IGF-1) for the treatment of Rett syndrome. Proc Natl Acad Sci U S A.

[32] Stary HC (1995). A definition of advanced types of atherosclerotic lesions and a histological classification of atherosclerosis. A report from the Committee on Vascular Lesions of the Council on Arteriosclerosis, American Heart Association. Circulation.

[33] Sukhanov S (2011). Differential requirement for nitric oxide in IGF-1-induced anti-apoptotic, anti-oxidant and anti-atherosclerotic effects. FEBS Lett.

[34] Chakraborty R (2021). Targeting smooth muscle cell phenotypic switching in vascular disease. JVS Vasc Sci.

[35] Kovacic JC (2019). Endothelial to mesenchymal transition in cardiovascular disease: JACC state-of-the-art review. J Am Coll Cardiol.

[36] Blackstock CD (2014). Insulin-like growth factor-1 increases synthesis of collagen type I via induction of the mRNA-binding protein LARP6 expression and binding to the 5’ stem-loop of COL1a1 and COL1a2 mRNA. J Biol Chem.

[37] Shai SY (2010). Smooth muscle cell-specific insulin-like growth factor-1 overexpression in Apoe-/- mice does not alter atherosclerotic plaque burden but increases features of plaque stability. Arterioscler Thromb Vasc Biol.

[38] Delafontaine P (2004). Expression, regulation, and function of IGF-1, IGF-1R, and IGF-1 binding proteins in blood vessels. Arterioscler Thromb Vasc Biol.

[39] Martinet W (2001). Oxidative DNA damage and repair in experimental atherosclerosis are reversed by dietary lipid lowering. Circ Res.

[40] Mah LJ (2010). gammaH2AX: a sensitive molecular marker of DNA damage and repair. Leukemia.

[41] Paffen E, DeMaat MP (2006). C-reactive protein in atherosclerosis: A causal factor?. Cardiovasc Res.

[42] Paul A (2004). C-reactive protein accelerates the progression of atherosclerosis in apolipoprotein E-deficient mice. Circulation.

[43] Snarski P (2022). Macrophage-specific IGF-1 overexpression reduces CXCL12 chemokine levels and suppresses atherosclerotic burden in Apoe-deficient mice. Arterioscler Thromb Vasc Biol.

[44] Kologrivova I (2020). Frequency of monocyte subsets is linked to the severity of atherosclerosis in patients with ischemic heart disease: a case-control study. Biomedicine (Taipei).

[45] Schroeder A (2006). The RIN: an RNA integrity number for assigning integrity values to RNA measurements. BMC Mol Biol.

[46] Butler A (2018). Integrating single-cell transcriptomic data across different conditions, technologies, and species. Nat Biotechnol.

[47] Dong R, Yuan GC (2021). SpatialDWLS: accurate deconvolution of spatial transcriptomic data. Genome Biol.

[48] Wirka RC (2019). Atheroprotective roles of smooth muscle cell phenotypic modulation and the TCF21 disease gene as revealed by single-cell analysis. Nat Med.

[49] Lafontaine DL, Tollervey D (2001). The function and synthesis of ribosomes. Nat Rev Mol Cell Biol.

[50] Tong W (2020). Foam cell-derived CXCL14 muti-functionally promotes atherogenesis and is a potent therapeutic target in atherosclerosis. J Cardiovasc Transl Res.

[51] Alonso A (2015). Molecular imaging of carotid plaque vulnerability. Cerebrovasc Dis.

[52] Perbal B (2013). CCN proteins: a centralized communication network. J Cell Commun Signal.

[53] To WS, Midwood KS (2011). Plasma and cellular fibronectin: distinct and independent functions during tissue repair. Fibrogenesis Tissue Repair.

[54] Zhao CF, Herrington DM (2016). The function of cathepsins B, D, and X in atherosclerosis. Am J Cardiovasc Dis.

[55] Eckert J (2015). Coronary CT angiography in managing atherosclerosis. Int J Mol Sci.

[56] Kolodgie FD (2003). Intraplaque hemorrhage and progression of coronary atheroma. N Engl J Med.

[57] Man JJ (2020). Sex as a biological variable in atherosclerosis. Circ Res.

[58] Link RP (1972). Effect of exercise on development of atherosclerosis in swine. Atherosclerosis.

[59] Kobari Y (1991). Regression of diet-induced atherosclerosis in Göttingen miniature swine. Lab Anim.

[60] Oppi S (2019). Mouse models for atherosclerosis research-which is my line?. Front Cardiovasc Med.

[61] Fan J (2015). Rabbit models for the study of human atherosclerosis: from pathophysiological mechanisms to translational medicine. Pharmacol Ther.

[62] Hajar R (2017). Risk factors for coronary artery disease: historical perspectives. Heart Views.

[63] Davignon J, Ganz P (2004). Role of endothelial dysfunction in atherosclerosis. Circulation.

[64] Sluiter TJ (2021). Endothelial barrier function and leukocyte transmigration in atherosclerosis. Biomedicines.

[65] Higashi Y (2020). Endothelial deficiency of insulin-like growth factor-1 receptor reduces endothelial barrier function and promotes atherosclerosis in Apoe-deficient mice. Am J Physiol Heart Circ Physiol.

[66] Bach LA (2015). Endothelial cells and the IGF system. J Mol Endocrinol.

[67] Higashi Y (2013). Insulin-like growth factor-1 regulates glutathione peroxidase expression and activity in vascular endothelial cells: Implications for atheroprotective actions of insulin-like growth factor-1. Biochim Biophys Acta.

[68] Sukhanov S (2015). Insulin-like growth factor I reduces lipid oxidation and foam cell formation via downregulation of 12/15-lipoxygenase. Atherosclerosis.

[69] Okura Y (2001). Decreased expression of insulin-like growth factor-1 and apoptosis of vascular smooth muscle cells in human atherosclerotic plaque. J Mol Cell Cardiol.

[70] Li Y (2003). Insulin-like growth factor-1 receptor activation inhibits oxidized LDL-induced cytochrome C release and apoptosis via the phosphatidylinositol 3 kinase/Akt signaling pathway. Arterioscler Thromb Vasc Biol.

[71] Depuydt MAC (2020). Microanatomy of the human atherosclerotic plaque by single-cell transcriptomics. Circ Res.

[72] Miao G (2022). Vascular smooth muscle cell c-Fos is critical for foam cell formation and atherosclerosis. Metabolism.

[73] Ye N (2014). Small molecule inhibitors targeting activator protein 1 (AP-1). J Med Chem.

[74] Cheng SM (2004). Irbesartan inhibits human T-lymphocyte activation through downregulation of activator protein-1. Br J Pharmacol.

[75] Kouzeli A (2020). CXCL14 preferentially synergizes with homeostatic chemokine receptor systems. Front Immunol.

[76] Ambrose JA (1988). Angiographic progression of coronary artery disease and the development of myocardial infarction. J Am Coll Cardiol.

[77] https://www.avma.org/sites/default/files/2020-02/Guidelines-on-Euthanasia-2020.pdf.

[78] Lystig TC (2003). Adjusted P values for genome-wide scans. Genetics.

