# Supplementary material for: Insulin-like growth factor 1 reduces coronary atherosclerosis in pigs with familial hypercholesterolemia
Source: JCI Insight. 2023 Feb 22;8(4):e165713. doi: 10.1172/jci.insight.165713 (PMC9990768; doi:10.1172/jci.insight.165713)
Supplement: Supplemental data [file jciinsight-8-165713-s011.pdf]

Extended methods

Intravascular ultrasound (IVUS). We used 14-month-old FH non-castrated males (N=5/group) and gilt (never been used for breeding) females (N=9/group) and administered recombinant human IGF-1 (rhIGF-1), 50 ug/kg/day, twice per day, or saline for 6 months. Intravascular ultrasound (IVUS) was performed in the RCA and LAD at basal level and after 3 months and 6 months of injections. We assigned 2 pigs per IVUS procedure/day: one pig from the IGF-1 group and one from the saline group. Pigs were pre-treated with a daily dose of aspirin (325 mg) for 3 days before IVUS. All animals were fasted for 12 hours prior to IVUS procedure. Telazol (2.25-6 mg/kg IM) and xylazine (1-2.25 mg/kg IM) were administered for anesthesia. An intravenous catheter was placed in a marginal ear vein for the administration of fluids and drugs. The auricular artery was canulated to allow for arterial blood pressure measurements and baseline blood pressure. Diazepam (0.5-1.0 mg/kg IV) was given to aid with intubation. An appropriately sized endotracheal tube was inserted for mechanical ventilation. Pigs were intubated and ventilated with oxygen mixed with nitrogen (compressed medical air). Ventilation parameters were set to maintain inspiratory pressures between 15–30 mmHg, respiration rate of 10–20 BPM and spO_2 92–100%. Arterial blood gases were taken and if pO_2, CO_2 and pH were out of the reference range, ventilation parameters were adjusted to correct. General anesthesia was maintained with isoflurane (1.5 to 3%) in O_2. Blood oxygenation was monitored using continuous pulse oximetry. Electrocardiogram was monitored throughout the entire procedure to detect myocardial infarction and possible arrhythmias requiring medical intervention. The pig was placed on a heating blanket to maintain rectal temperature at 36.5 to 39.0°C (assessed via rectal probe). Artificial tears ophthalmic ointment was applied bilaterally to the corneal surfaces to keep the corneas moist during the procedure. After intubation and clipping the vascular access area, a sterile prep was performed using 3 cycles of surgical scrub betadine or chlorohexidine. The right or left femoral artery and vein were cannulated with a percutaneous sheath for catheter advancement into the coronary arteries. To prevent thrombosis throughout the duration of the procedure, a loading dose of heparin was administered as needed to achieve an activated clotting time >300 seconds. Contrast-enhanced coronary cine angiographic images were acquired and stored using the Innova Optima CL323i X-ray

system (GE Healthcare, Pewaukee, WI). Intravascular ultrasound was performed using the IVUS imaging system (Volcano Corporation, San Diego, CA) and a 20 MHz 3.5 F Visions PV 0.035 Digital IVUS Catheter (Volcano Corporation, San Diego, CA) advanced to the left anterior descending artery (LAD) and the right coronary artery (RCA) through a standard 5 F guide catheter. Operative management included fluid maintenance (0.9% saline) and the administration of long-acting cefazolin sodium (15 mg/kg, IM) as antibiotic prophylaxis. After the procedure, the catheters were removed, and the artery closed (Angio-Seal Vascular Closure Device, Terumo Medical Corp, New Jersey, NJ). Analgesia was provided with Buprenex-SR (0.12-0.27 mg/kg SC. Following the final invasive imaging procedure, pigs were euthanized while under deep isoflurane anesthesia using potassium chloride (KCl, 20 mEq IV) to arrest the heart in diastole.

To inject solutions, we used a Stratis needle-free injection system (PharmaJet, Golden, CO). All pigs received a high-fat diet (HFD) starting the day after the T0 IVUS. The HFD contains 800 g/day (by weight 13% protein, 39.6% carbohydrate, 47.4% fat and 2% cholesterol)(1). Pigs have free access to water. Body weight was measured weekly.

IVUS analysis. Coronary arteries contain the tunica intima layer (a complex of endothelium, atheroma, and internal elastic membrane (IEM)), tunica media layer (TM) (TM consists of smooth muscle and external elastic membrane (EEM)) and tunica externa layer which comprises the tunica adventitia (TA) and peri-adventitial tissues(2). Since the leading edge of the media (corresponding to the IEM) is not well delineated, IVUS measurements cannot determine true histological atheroma area. Consistent with the 2021 clinical expert consensus document on IVUS measurements the EEM and lumen areas are used to calculate a surrogate for true atherosclerotic plaque area and the term "plaque plus media area" is recommended(2). For the current study 20 mm of IVUS pullback segment distal to the ostia of the right coronary artery (RCA) and the left anterior descending artery (LAD) were selected. The area circumscribed by the outer border of the echolucent TM and the luminal border was manually traced on each 1 mm IVUS frame within selected fragment. SS and ZR independently performed the manual outline of both areas, and ZR was blinded to the individual animal assignment. The inter-rater variability

was 10.5%, 7.2% and 10.8% for evaluation of lumen volume, vessel volume and relative atheroma volume, respectively.

Atherosclerotic burden assessment by histology. Following completion of the experimental protocol, hearts were excised in accordance with the recommendation of the American Veterinary Medical Association Guide on Euthanasia (2020 Edition). The entire right coronary (RCA) and the left anterior descending (LAD) arteries were collected, and the proximal 30 mm fragment of RCA and LAD was immersed into 10% formalin, and further cut onto 6x5 mm fragments. One Trichrome-stained section per each block was used to quantify atherosclerotic burden. Sections were imaged with Olympus IX71 inverted microscope equipped with DP80 camera. Analysis of vessel morphometry was performed with CellSens Dimension 1.18 software (Olympus) by two independent researchers, one of them blinded. The mean±SEM discrepancy between evaluators was 11.2±1.7%. EEM, IEM and luminal border were manually outlined, and corresponding cross-sectional areas (CSA) were measured.

The following indices were assessed:

1) relative tunica media CSA = (EEM-IEM)/EEM x 100%.
2) relative atherosclerotic plaque CSA = (IEM-Luminal area)/EEM x 100%.
3) necrotic core (NC) area and fibrous cap (FC) area were manually outlined using CellSens Dimension software. The NC was defined as acellular (hematoxylin-negative) plaque area.
4) FC was defined as largely uninterrupted strip of brown-colored (smooth muscle, connective tissues) material on the top of necrotic or lipid core with a higher density of nuclei than the plaque core. The thickness of FC was calculated as the mean length of 5 arbitrary lines distributed across the cap area.

Plaque composition analysis. The cellular content of coronary plaques was assessed by immunohistochemistry (IHC). To perform IHC coronary sections were deparaffinized, dehydrated and processed with heat-mediated antigen retrieval using citrate buffer (pH 6.0) followed by blocking step (Protein block, Abcam, ab64226). Sections were incubated overnight at +4°C with mixture of primary antibodies, or with mixture of normal IgG (negative control). The 1st section was incubated with mouse α-SMA antibody (Millipore,

CBL171, clone ASM1) plus rabbit pH2A.X histone antibody (Cell Signaling, 9718, clone 20E3). The 2nd serial section was incubated with mouse SRA antibody (TransGenia Inc., KT022, clone SRA-E5) plus rabbit CD31 antibody (Abcam, 134168, clone EP3095) and the 3rd serial section with mouse IgG plus rabbit IgG (both are from Santa Cruz Biotechnology, sc-2025 and sc-2027, respectively). Rabbit antibody signal was visualized with goat anti-rabbit-biotin IgG (Abcam, BA-1000) followed by incubation with streptavidin-AlexaFluor594 conjugate (Life Technologies, S32356) plus DAPI. Mouse antibody signal was amplified with Alexa Fluor 488 Tyramide Super Boost kit (Invitrogen, B40912). Sections were mounted with ProLong Gold antifade media (Thermo Fisher, P36970) for imaging. To quantify immunopositivity for each antibody, sections were scanned with Cytation 5 multi-mode imager (Bio-Tek, Winooski, MI) using standard Texas Red, GFP and DAPI filter cubes to generate greyscale images for each channel. Next, vessel morphological regions-of-interest (ROI) (i.e., TM, plaque, etc.) were manually outlined and area positive for cellular marker was quantified within ROI. The ratio of marker-positive area per ROI area (x 100%) was calculated and shown in Figures.

Cell apoptosis. To quantify cell apoptosis, we used *In Situ* Cell Death Detection Kit, TMR red (Millipore, 12156792910**)** as per manufacturer's instructions. Briefly, sections were deparaffinized, dehydrated and permeabilized with Digest-All 2 (Trypsin) kit (Invitrogen, OO3008). Next, sections were incubated with TUNEL mixture diluted 1:2 with TUNEL dilution buffer (Roche, 11966006001) for 45 min at 37°C, washed with PBS, co-stained with DAPI and mounted with ProLong Gold antifade media. Treatment with DNase I (Ambion, AM2222, 10U/sections) for 20 min at 37°C was used to generate positive control. Each slide with TUNEL-stained section contained a serial section stained with dUTP-omitted TUNEL mixture serving as a negative control. Total cell apoptosis was defined as TUNEL-positive cell number per 1000 DAPI-positive nuclei.

Monocyte subsets. To count circulating CD163^{lo}/CD14^{hi} monocytes, whole blood was mixed with a cocktail of monoclonal antibodies against CD163-PE (1: 80 dilution, Bio-Rad, MCA2311PE), and CD14-Alexa Fluor 488 (1: 20 dilution, Bio-Rad, MCA1568A488), porcine CD172a (1: 100 dilution, Southern Biotech, 4525-08), or appropriate isotype controls and subsequently with streptavidin-APC/Cy7 (1: 1000 dilution, Southern Biotech,

7105-19). Red blood cells were lysed by BD Pharm Lyse lysing solution (BD Biosciences, 555899) and washed 3 times using PBS before flow-cytometric analysis. Data acquisition and analysis were performed using Beckman Coulter Epics Gallios analyzer running Gallios software v1.1; CD172a-positive leukocytes were further size-gated to identify monocytes. The monocyte-gate was differentiated into two subsets based on CD163 and CD14 expression levels and counted as CD163^{lo}/CD14^{hi} monocytes and CD163^{hi}/CD14^{lo} monocytes(3).

Spatial transcriptomics (ST). ST was conducted using the Visium Spatial Gene Expression System (10× Genomics, Pleasanton, CA). The RCA fragments were snap-frozen, embedded with OCT compound and cut at –26°C at a thickness of 10 µm. Optimization and gene expression assays were carried out according to the manufacturer's protocol. Briefly, slides were fixed in –20°C methanol, dried with isopropanol, and stained with H&E. A tile scan image of the entire section was generated using a Cytation 5 imager. For tissue optimization, enzymatic permeabilization was conducted for 0–40 min, followed by first-strand cDNA synthesis with fluorescent nucleotides. The slide was reimaged using standard RFP filter cube. An optimal permeabilization time of 12 min was determined by visual inspection to maximize mRNA recovery while at the same time minimizing diffusion. Library preparation, clean-up, and indexing were conducted using company manual-guided procedures. Samples were subjected to pair-ended sequencing using an Illumina NextSeq 2000 generating ~800 M reads. Pig reference genome was created from *Sus scrofa* genomic sequence (Sscrofa 11.1) and Ensembl annotation, and reads were aligned and counted by Space Ranger (10x Genomics). All the downstream analyses were performed using R toolkit Seurat(4) and Ingenuity pathway analysis (IPA) software (Qiagen). SMC-, MF- and fibromyocytes (FM) distribution and calculation of cell type ratio were predicted by mixed cell deconvolution analysis driven by Seurat using human atherosclerotic RCA single cell RNA seq dataset (accession number GSE131778)(5).

References

1. Masseau I, and Bowles DK. Carotid Endothelial VCAM-1 Is an Early Marker of Carotid Atherosclerosis and Predicts Coronary Artery Disease in Swine. *J Biomed Sci Eng.* 2015;8(11):789-96.

2. Saito Y, Kobayashi Y, Fujii K, Sonoda S, Tsujita K, Hibi K, et al. Clinical expert consensus document on intravascular ultrasound from the Japanese Association of Cardiovascular Intervention and Therapeutics (2021). *Cardiovasc Interv Ther.* 2022;37(1):40-51.
3. Gordon S, and Taylor PR. Monocyte and macrophage heterogeneity. *Nat Rev Immunol.* 2005;5(12):953-64.
4. Butler A, Hoffman P, Smibert P, Papalexi E, and Satija R. Integrating single-cell transcriptomic data across different conditions, technologies, and species. *Nat Biotechnol.* 2018;36(5):411-20.
5. Wirka RC, Wagh D, Paik DT, Pjanic M, Nguyen T, Miller CL, et al. Atheroprotective roles of smooth muscle cell phenotypic modulation and the TCF21 disease gene as revealed by single-cell analysis. *Nat Med.* 2019;25(8):1280-9.

Supplemental Table 1. FH pigs blood pressure (BP) and heart rate (HR)

Time-point/drug	Sex	BP, mmHg, systolic		BP, mmHg, diastolic		HR, bpm	
		**Mean**	**SEM**	**Mean**	**SEM**	**Mean**	**SEM**
T0	M	109	5.3	83.6	6.7	93.6	7.8
	F	114.5	6.6	76.3	4.5	102.7	5.8
T3/Saline	M	118.0	6.6	91.0	3.8	104.3	7.3
	F	130.7	14.5	87.2	8.0	94.0	12.9
T3/IGF-1	M	129.3	4.5	85.5	4.5	102.2	4.4
	F	121.8	7.6	81.0	4.0	104.7	9.8
T6/Saline	M	127.0	9.1	84.0	7.5	100.5	8.6
	F	133.7	3.7	91.8	8.6	89.0	3.9
T6/IGF-1	M	120.2	7.8	72.0	7.1	99.3	6.1
	F	116.5	7.3	68.8	4.8	100.2	6.7

Supplemental Table 2A. FH pigs blood biochemistry (males).

Male			0		1-mo		2-mo		3-mo		4-mo		5-mo		6-mo	
	Units	Group	Avg	SD	Avg	SD	Avg	SD	Avg	SD	Avg	SD	Avg	SD	Avg	SD
Total Protein	g/dL	Saline	6.5	0.4	6.7	0.3	6.3	0.4	5.8	0.3	6.2	0.3	6.4	0.4	6.5	0.4
		IGF-1	6.4	0.3	6.4	0.1	6.0	0.3	5.9	0.3	6.1	0.4	6.3	0.3	6.2	0.4
Albumin	g/dL	Saline	4.1	0.1	4.2	0.1	4.1	0.2	3.9	0.2	4.1	0.3	4.0	0.5	4.0	0.4
		IGF-1	4.0	0.2	3.8	0.1	3.5	0.1	3.5	0.1	3.7	0.2	3.9	0.2	3.7	0.2
Globulin	g/dL	Saline	2.4	0.4	2.5	0.3	2.2	0.5	1.9	0.4	2.1	0.3	2.3	0.6	2.5	0.7
		IGF-1	2.4	0.3	2.6	0.2	2.4	0.3	2.4	0.3	2.4	0.3	2.5	0.3	2.5	0.3
A/G Ratio		Saline	1.8	0.4	1.7	0.2	2.0	0.4	2.1	0.5	2.0	0.4	1.9	0.7	1.7	0.6
		IGF-1	1.7	0.3	1.5	0.2	1.5	0.2	1.5	0.2	1.6	0.3	1.6	0.3	1.5	0.3
AST (SGOT)	IU/L	Saline	24	2.9	39	17.0	50	64.6	22	3.3	24	3.8	26	9.4	20	2.5
		IGF-1	30	12.4	25	4.3	24	6.1	23	6.6	22	6.0	21	3.1	28	18.4
ALT (SGPT)	IU/L	Saline	28	0.8	29	3.0	28	5.5	23	2.4	22	2.4	20	3.3	21	2.3
		IGF-1	33	6.6	32	2.8	27	2.9	24	2.0	23	1.6	21	2.3	27	9.8
Alk Phosphatase	IU/L	Saline	78	30	96	39	95	44	81	38	93	38	75	28	83	41
		IGF-1	80	22	107	23	90	17	79	21	80	22	84	23	82	37
GGT	IU/L	Saline	28	9	32	10	28	9	28	9	32	10	34	8	31	8
		IGF-1	34	9	37	8	34	7	32	7	33	5	37	7	38	5
Total Bilirubin	mg/dL	Saline	0.1	0.0	0.2	0.0	0.1	0.0	0.2	0.1	0.1	0.0	0.1	0.0	0.1	0.0
		IGF-1	0.1	0.0	0.1	0.1	0.1	0.0	0.2	0.0	0.1	0.1	0.1	0.0	0.1	0.0
BUN	mg/dL	Saline	10	1.8	9	1.6	10	0.8	9	2.2	9	1.5	12	2.6	13	1.8
		IGF-1	9	1.3	8	1.8	8	2.0	8	1.7	7	1.5	9	1.9	12	1.8
Creatinine	mg/dL	Saline	1.5	0.1	1.5	0.1	1.6	0.2	1.6	0.1	1.4	0.2	1.5	0.1	1.5	0.2
		IGF-1	1.4	0.1	1.4	0.2	1.5	0.1	1.4	0.2	1.3	0.1	1.4	0.1	1.4	0.2
BUN/CREAT RATIO		Saline	7	1.3	6	0.9	6	0.0	6	0.9	7	1.3	8	1.6	8	1.6
		IGF-1	7	1.5	6	1.1	5	1.7	6	1.8	6	1.3	6	1.2	9	0.5
Phosphorus	mg/dL	Saline	6.9	0.7	6.1	0.5	6.5	0.5	5.6	0.3	5.5	0.1	5.7	0.3	6.0	0.5
		IGF-1	7.4	0.9	6.5	0.8	5.7	0.8	6.2	0.8	5.8	0.7	6.3	0.4	6.3	0.6
Glucose	mg/dL	Saline	156	80	79	12	87	9	180	41	92	7	100	16	132	39
		IGF-1	90	16	83	7	117	23	108	44	90	9	116	45	133	11
CALCIUM	mg/dL	Saline	10.7	0.5	10.8	0.2	10.7	0.2	10.6	0.2	10.6	0.3	10.5	0.5	10.7	0.2
		IGF-1	10.6	0.5	10.5	0.2	10.2	0.1	10.3	0.5	10.3	0.1	10.3	0.3	10.2	0.3

Supplemental Table 2B. FH pigs blood biochemistry (males, continuation).

Male			0		1-mo		2-mo		3-mo		4-mo		5-mo		6-mo	
	Units	Group	Avg	SD	Avg	SD	Avg	SD	Avg	SD	Avg	SD	Avg	SD	Avg	SD
Magnesium	mEq/L	Saline	2.0	0.3	1.9	0.2	2.0	0.2	1.9	0.1	2.0	0.2	1.9	0.1	2.0	0.2
		IGF-1	1.9	0.2	1.9	0.2	1.8	0.1	1.8	0.2	1.9	0.1	2.0	0.2	2.0	0.2
Sodium	mEq/L	Saline	144	3	143	1	144	1	142	2	144	1	141	2	144	2
		IGF-1	143	2	141	4	143	1	143	3	144	2	141	3	144	1
Potassium	mEq/L	Saline	4.0	0.3	3.9	0.1	4.4	0.5	4.2	0.2	4.1	0.3	4.2	0.2	4.6	0.1
		IGF-1	4.0	0.5	4.1	0.3	3.9	0.1	4.0	0.3	4.1	0.3	4.3	0.8	4.6	0.5
NA/K RATIO		Saline	36	3.5	36	1.3	33	3.4	34	2.1	35	2.1	34	1.5	32	1.3
		IGF-1	36	3.6	34	2.2	36	1.5	36	2.4	35	2.1	33	5.3	32	2.8
Chloride	mEq/L	Saline	99	4	99	2	99	1	100	1	103	2	101	2	102	1
		IGF-1	97	3	100	3	100	2	100	2	101	2	100	3	101	2
Cholesterol	mg/dL	Saline	150	42	361	83	366	62	331	52	307	48	289	131	250	56
		IGF-1	163	51	415	106	410	111	347	94	305	107	344	128	282	102
TRIGLYCERIDE	mg/dL	Saline	32	8	28	4	61	20	42	12	40	6	39	19	47	17
		IGF-1	33	14	37	23	29	10	30	5	32	5	34	8	41	7
Amylase	IU/L	Saline	1418	360	1603	360	1559	350	1441	343	1593	399	1546	411	1523	372
		IGF-1	1374	312	1459	340	1429	350	1381	379	1478	446	1501	416	1399	376
PrecisionPSL	U/L	Saline	7	1	7	1	8	2	6	1	6	2	6	1	7	1
		IGF-1	8	1	9	5	7	0	6	1	6	0	6	1	6	1
CPK	IU/L	Saline	408	121	2154	2160	3519	6915	498	112	621	359	753	914	381	54
		IGF-1	798	649	701	673	616	263	538	299	573	358	345	100	1715	3065

Supplemental Table 2C. FH pigs blood biochemistry (females)

Female			0		1-mo		2-mo		3-mo		4-mo		5-mo		6-mo	
	Units	Group	Avg	SD	Avg	SD	Avg	SD	Avg	SD	Avg	SD	Avg	SD	Avg	SD
Total Protein	g/dL	Saline	6.6	0.5	6.7	0.5	6.6	0.4	6.6	0.3	6.9	0.5	7.0	0.6	6.8	0.3
		IGF-1	6.5	0.3	6.5	0.4	6.2	0.5	6.1	0.3	6.5	0.2	6.6	0.8	6.3	0.6
Albumin	g/dL	Saline	3.8	0.5	3.7	0.4	3.9	0.4	3.7	0.3	3.8	0.4	4.0	0.3	3.8	0.5
		IGF-1	3.8	0.2	3.6	0.3	3.9	0.2	3.7	0.3	4.1	0.3	3.8	0.6	3.6	0.4
Globulin	g/dL	Saline	2.8	0.3	2.9	0.6	2.6	0.5	2.9	0.5	3.1	0.6	3.1	0.6	3.0	0.4
		IGF-1	2.7	0.4	2.9	0.7	2.4	0.6	2.4	0.5	2.4	0.3	2.8	1.4	2.7	0.9
A/G Ratio		Saline	1.4	0.3	1.3	0.3	1.5	0.3	1.3	0.2	1.3	0.3	1.3	0.3	1.3	0.4
		IGF-1	1.5	0.4	1.3	0.4	1.7	0.5	1.6	0.5	1.7	0.3	1.6	0.7	1.5	0.5
AST (SGOT)	IU/L	Saline	18	4.3	20	2.4	20	1.5	20	2.1	20	3.8	28	12.3	20	7.0
		IGF-1	20	2.9	20	3.4	25	11.7	20	2.7	38	30.1	20	2.6	19	2.3
ALT (SGPT)	IU/L	Saline	25	3.0	27	3.4	27	3.4	23	3.3	21	1.3	21	2.3	21	3.2
		IGF-1	23	3.4	25	1.8	25	1.9	22	2.3	24	4.4	21	2.9	21	2.7
Alk Phosphatase	IU/L	Saline	65	15	75	23	69	19	64	21	60	23	67	18	61	15
		IGF-1	68	19	79	18	73	13	74	19	69	16	64	16	59	13
GGT	IU/L	Saline	25	6	26	9	24	9	37	13	25	9	28	8	28	7
		IGF-1	26	12	24	8	21	10	34	24	27	12	23	9	23	10
Total Bilirubin	mg/dL	Saline	0.1	0.1	0.1	0.0	0.1	0.0	0.1	0.1	0.1	0.0	0.1	0.0	0.1	0.0
		IGF-1	0.2	0.1	0.1	0.1	0.1	0.0	0.1	0.1	0.1	0.0	0.1	0.0	0.1	0.0
BUN	mg/dL	Saline	11	4.8	11	1.5	12	1.8	12	3.1	9	1.9	10	1.7	12	2.3
		IGF-1	10	1.9	8	1.5	7	0.7	7	1.1	8	1.8	7	1.5	8	1.5
Creatinine	mg/dL	Saline	1.5	0.2	1.6	0.1	1.5	0.2	1.7	0.2	1.8	0.2	1.8	0.2	1.8	0.3
		IGF-1	1.5	0.2	1.3	0.2	1.4	0.2	1.5	0.2	1.6	0.2	1.5	0.2	1.5	0.2
BUN/CREAT RATIO		Saline	8	3.9	7	1.0	8	1.9	7	1.9	5	1.7	6	1.3	7	2.1
		IGF-1	7	2.1	6	1.1	5	0.8	5	0.4	5	1.3	5	0.5	5	0.8
Phosphorus	mg/dL	Saline	5.8	0.3	5.7	0.2	5.8	0.2	5.8	0.8	5.6	0.4	5.6	0.4	5.8	0.7
		IGF-1	5.8	0.1	6.4	0.5	5.7	0.3	5.7	0.6	5.9	0.5	5.5	0.6	5.4	0.5
Glucose	mg/dL	Saline	141	9	112	24	101	14	146	54	112	33	91	10	113	13
		IGF-1	128	23	93	11	100	21	104	29	100	12	84	13	86	11
CALCIUM	mg/dL	Saline	10.8	0.5	9.9	0.3	10.4	0.4	10.4	0.2	10.4	0.3	10.5	0.2	10.4	0.3
		IGF-1	10.7	0.2	9.9	0.4	10.2	0.2	10.3	0.1	10.3	0.2	10.1	0.3	9.8	0.4

Supplemental Table 2D. FH pigs blood biochemistry (females, continuation).

Female			0		1-mo		2-mo		3-mo		4-mo		5-mo		6-mo	
	Units	Group	Avg	SD	Avg	SD	Avg	SD	Avg	SD	Avg	SD	Avg	SD	Avg	SD
Magnesium	mEq/L	Saline	2.0	0.2	2.0	0.3	2.1	0.3	1.9	0.3	1.9	0.2	1.9	0.2	2.0	0.1
		IGF-1	1.8	0.1	1.8	0.2	1.8	0.1	1.7	0.1	1.8	0.2	1.6	0.1	1.6	0.1
Sodium	mEq/L	Saline	141	3	140	2	138	3	135	4	139	3	138	2	139	4
		IGF-1	140	1	140	2	139	0	140	2	141	2	140	1	139	2
Potassium	mEq/L	Saline	4.0	0.1	4.0	0.2	4.1	0.6	4.2	0.4	4.3	0.6	3.9	0.2	4.2	0.5
		IGF-1	3.9	0.1	4.3	0.3	4.0	0.3	4.0	0.1	4.7	0.9	4.2	0.3	4.1	0.2
NA/K RATIO		Saline	35	1.2	35	2.5	34	4.1	32	2.9	33	4.1	36	1.5	34	3.8
		IGF-1	36	1.3	33	2.5	35	2.5	35	0.8	31	5.4	34	2.2	34	1.9
Chloride	mEq/L	Saline	99	3	100	2	97	3	94	3	99	3	98	3	98	3
		IGF-1	99	2	99	2	100	1	101	1	101	2	101	1	102	1
Cholesterol	mg/dL	Saline	441	153	932	337	917	359	922	428	812	329	860	367	809	293
		IGF-1	427	95	696	96	764	196	764	173	723	181	655	220	615	139
TRIGLYCERIDE	mg/dL	Saline	83	26	54	13	61	10	61	10	57	17	63	25	91	31
		IGF-1	55	22	48	35	45	11	40	14	49	19	48	35	38	8
Amylase	IU/L	Saline	1199	267	1188	479	1192	464	1154	420	1128	387	1284	546	1271	475
		IGF-1	1283	502	1313	467	1355	514	1287	504	1283	448	1340	537	1378	561
Precision PSL	U/L	Saline	10	5	8	1	8	2	8	1	8	1	8	2	9	1
		IGF-1	8	0	8	2	7	0	7	0	9	3	7	1	9	1
CPK	IU/L	Saline	260	74	286	33	343	95	338	90	408	345	919	985	282	61
		IGF-1	509	412	347	155	874	1245	330	74	1405	2095	603	818	271	91

Supplemental Table 3A. FH pigs CBC with differential (males)

Male			0		1-mo		2-mo		3-mo		4-mo		5-mo		6-mo	
**Complete Blood Count**	**Units**	**Group**	**Avg**	**SD**	**Avg**	**SD**	**Avg**	**SD**	**Avg**	**SD**	**Avg**	**SD**	**Avg**	**SD**	**Avg**	**SD**
WBC	10^3/uL	Saline	9.2	2.4	6.7	1.0	7.4	0.7	7.4	1.3	8.0	2.0	7.9	1.4	8.6	1.6
		IGF-1	9.1	2.8	8.0	1.2	7.4	0.7	8.9	1.1	7.9	1.0	7.7	1.5	8.6	0.9
RBC	10^6/uL	Saline	6.0	1.6	6.1	0.7	5.6	0.5	4.8	0.5	5.1	0.5	4.9	0.4	4.7	0.5
		IGF-1	5.3	0.8	6.1	0.9	5.0	0.7	4.4	0.5	4.9	0.5	4.6	0.4	4.2	0.6
HGB	g/dL	Saline	11.4	2.7	12.0	1.3	11.2	0.9	10.0	1.1	10.5	1.1	10.4	0.8	9.8	1.0
		IGF-1	10.2	1.5	11.9	1.5	10.1	1.0	8.9	0.7	10.0	0.7	9.9	0.9	8.9	1.3
HCT	%	Saline	38	9	38	4	37	3	30	3	34	3	33	2	31	3
		IGF-1	33	5	38	5	31	4	27	3	33	3	31	3	29	4
MCV	fL	Saline	64	3	63	1	66	2	63	6	66	2	67	1	65	3
		IGF-1	63	4	62	2	63	2	62	6	67	1	67	2	68	2
MCH	pg	Saline	19.2	0.8	19.7	0.7	20.0	0.8	20.8	0.7	20.5	0.7	21.2	0.6	20.8	0.9
		IGF-1	19.1	0.8	19.7	0.9	20.7	1.1	20.2	1.6	20.5	0.7	21.5	1.0	21.3	0.9
MCHC	g/dL	Saline	30	0.9	31	0.4	30	0.5	33	2.4	31	0.4	32	1.1	32	2.1
		IGF-1	30	0.7	32	0.5	33	1.4	33	4.1	30	0.5	32	0.8	31	0.8
Platelet Count	10^3/uL	Saline	382	66	326	48	371	31	340	65	301	23	349	55	386	74
		IGF-1	346	65	253	41	274	104	258	86	324	74	289	43	305	79
Neutrophils	/uL	Saline	3790	2260	2105	780	2608	751	2750	1045	3346	1457	3239	1126	3183	1680
		IGF-1	4448	2162	3163	1291	2616	535	3594	308	3700	1259	3393	1165	3245	661
Lymphocytes	/uL	Saline	5017	757	4306	350	4518	559	4189	536	4239	875	4267	644	5140	1062
		IGF-1	4261	1521	4366	787	4516	774	4767	834	3618	640	3967	503	4700	127
Monocytes	/uL	Saline	352	109	254	62	284	50	321	114	415	265	295	61	170	151
		IGF-1	321	82	355	252	244	110	383	176	541	228	307	59	293	120
Eosinophils	/uL	Saline	50	47	55	32	30	41	120	51	39	54	99	66	106	56
		IGF-1	110	53	77	59	64	71	156	64	80	96	0	0	322	346
Basophils	/uL	Saline	0	0	0	0	0	0	0	0	0	0	0	0	0	0
		IGF-1	0	0	0	0	0	0	0	0	0	0	12	28	0	0

Supplemental Table 3B. FH pigs CBC with differential (females)

Female			0		1-mo		2-mo		3-mo		4-mo		5-mo		6-mo	
**Complete Blood Count**	**Units**	**Group**	**Avg**	**SD**	**Avg**	**SD**	**Avg**	**SD**	**Avg**	**SD**	**Avg**	**SD**	**Avg**	**SD**	**Avg**	**SD**
WBC	10^3/uL	Saline	7.1	3.3	6.2	1.8	6.9	2.5	6.4	2.0	7.1	3.4	7.6	2.9	6.7	3.0
		IGF-1	5.9	0.6	6.8	0.9	6.4	1.2	6.3	1.3	7.2	0.8	7.7	1.9	6.9	1.9
RBC	10^6/uL	Saline	4.4	0.4	5.0	0.7	4.8	0.6	4.6	0.4	4.7	0.7	5.3	0.9	4.6	0.6
		IGF-1	4.8	0.3	4.8	0.4	5.1	0.7	4.6	0.3	4.9	0.5	4.6	0.7	4.3	0.2
HGB	g/dL	Saline	8.4	0.4	9.3	1.0	9.2	0.8	8.9	0.8	9.2	1.3	10.3	1.3	8.9	0.9
		IGF-1	9.1	0.6	9.3	0.9	10.0	1.4	9.2	0.6	9.9	1.2	9.3	1.3	8.7	0.4
HCT	%	Saline	26	2	29	3	28	3	26	2	28	4	31	4	27	3
		IGF-1	28	3	29	3	30	4	27	2	30	3	28	4	26	2
MCV	fL	Saline	59	4	58	2	58	3	57	3	59	3	59	3	60	4
		IGF-1	59	4	60	3	59	1	59	1	61	1	61	3	62	1
MCH	pg	Saline	19.0	1.0	18.7	1.3	19.5	1.3	21.5	5.1	19.4	1.1	19.4	1.0	19.7	1.5
		IGF-1	18.9	0.5	19.5	0.7	19.6	0.8	20.1	0.5	20.4	0.5	20.2	0.5	20.1	0.4
MCHC	g/dL	Saline	33	2.6	32	1.6	34	0.8	34	0.8	33	0.4	33	0.4	33	0.8
		IGF-1	32	2.1	33	0.8	33	1.4	34	0.5	33	0.4	34	1.0	33	0.9
Platelet Count	10^3/uL	Saline	325	37	347	62	320	87	368	83	375	143	327	68	318	60
		IGF-1	290	110	345	112	280	99	286	95	257	139	243	172	282	122
Neutrophils	/uL	Saline	2904	2995	2366	1977	2477	2385	2454	2213	2802	2908	2906	2846	2509	2457
		IGF-1	1744	513	2489	1232	1535	1034	1455	900	1879	387	2466	1618	2234	1589
Lymphocytes	/uL	Saline	3985	1674	3666	1076	4174	645	3597	774	3930	809	4148	846	3740	520
		IGF-1	3683	778	3845	715	4455	1097	4506	754	4869	989	4737	1014	4407	467
Monocytes	/uL	Saline	147	40	167	89	185	129	272	80	270	93	367	99	292	123
		IGF-1	319	205	384	167	416	162	247	154	234	81	361	218	182	68
Eosinophils	/uL	Saline	84	80	0	0	24	54	56	61	79	52	139	109	91	42
		IGF-1	109	84	41	93	14	31	77	39	178	164	136	144	76	67
Basophils	/uL	Saline	0	0	0	0	0	0	0	0	0	0	0	0	47	106
		IGF-1	25	35	0	0	0	0	14	32	0	0	0	0	0	0

Supplementary Table 4. Quality controls for spatial transcriptomic (ST) analysis

**Injection protocol**	**IGF-1**	**IGF-1**	**Saline**	**Saline**	
**Quality controls/pigs ID**	**#651**	**#493**	**#654**	**#495**	**Mean±SEM**
Number of reads, millions	138.9	150.9	87.3	106.6	121.0±14.6
Median genes per Spot	1822.5	2181.0	2037.0	2449.0	2122.4±131.4
Median UMI counts per spot	5488.0	6914.0	5377.0	8131.0	6477.5±652.9
Valid barcodes, %	98.1	98.1	97.9	98.1	98.0±0.1
Valid UMIs, %	100.0	100.0	99.8	100.0	99.9±0.01
Q30 bases in barcode, %	96.0	96.2	96.0	95.9	96.0±0.1
Q30 bases in RNA read, %	95.7	95.7	95.5	95.7	95.6±0.01
Q30 bases in UMI, %	96.5	96.6	96.4	96.4	96.5±0.1
Reads mapped to genome, %	94.1	95.2	91.8	95.7	94.2±0.9
Reads mapped confidently to genome, %	90.8	93.1	89.2	93.6	91.7±1.0
Reads mapped confidently to intergenic regions, %	2.8	2.9	3.5	2.8	3.0±0.2
Reads mapped confidently to intronic regions, %	5.7	5.0	7.1	5.2	5.7±0.5
Reads mapped confidently to exonic regions, %	82.3	85.2	78.6	85.7	83.0±1.6
Reads mapped confidently to transcriptome, %	76.5	79.8	72.6	79.9	77.2±1.7
Reads mapped antisense to gene, %	1.0	1.1	1.4	1.1	1.2±0.1
Fraction reads in spots under tissue, %	85.7	80.2	83.4	81.6	82.7±1.2
Total genes detected	17937.0	16888.0	17129.0	16630.0	17146±282

Suppl.Fig.1

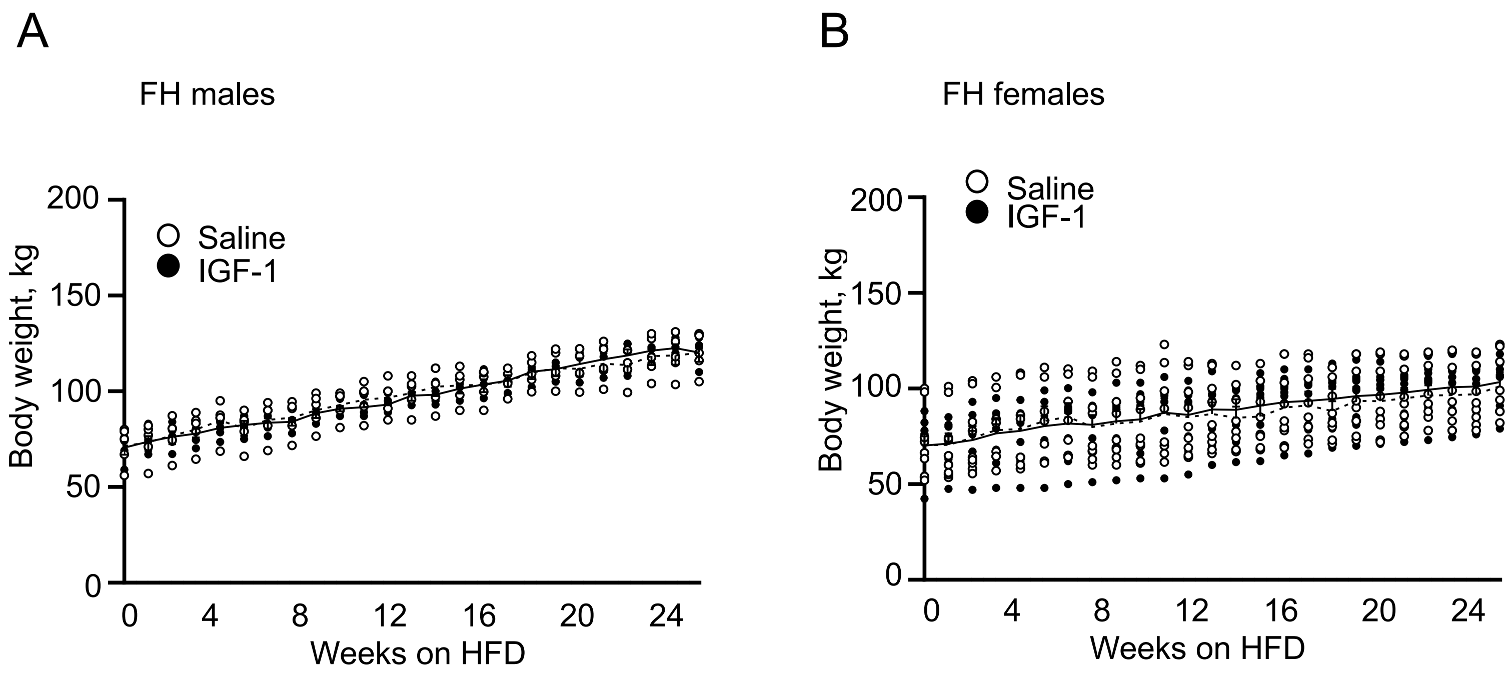

<u>Supplemental Figure 1.</u> FH pigs body weight. IGF-1 or saline (control) was injected into FH pigs (A, males, 5/group, B, females, 9/group) and pigs were fed with high-fat diet (HFD) for 6 months. Body weight was measured weekly. Individual data-points are shown (saline, empty circle, IGF-1 solid circle).

Suppl.Fig.2A

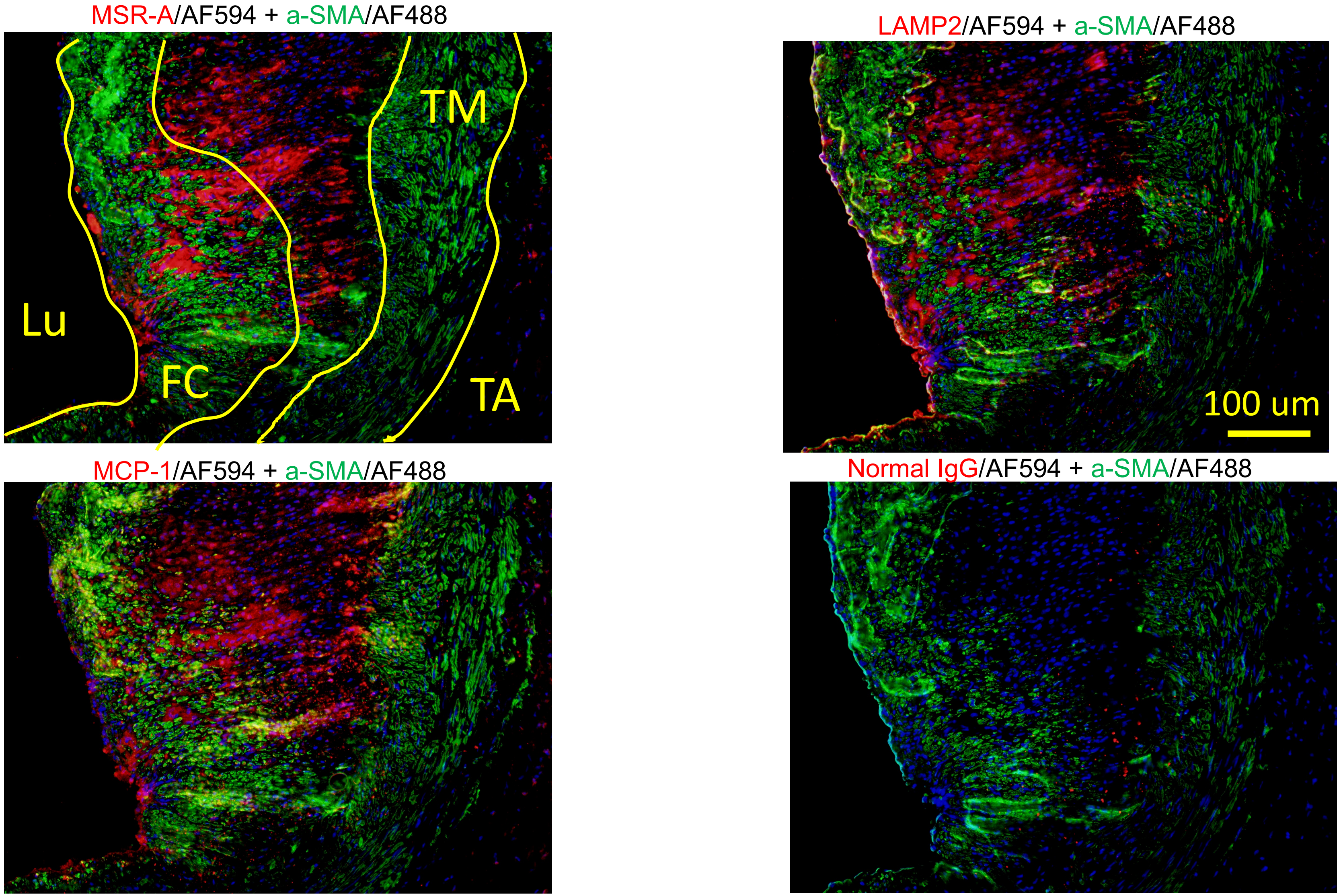

Supplemental Figure 2A. Validation of cell marker antibodies for immunohistochemistry. RCA serial cross-sections were obtained from saline-injected FH females. Sections were immunostained with antibody for macrophage scavenger receptor A (MSR-A), monocyte chemoattractant protein 1 (MCP-1), lysosome-associated membrane protein 2 (LAMP2) (all are macrophage markers) and co-stained with α-smooth muscle actin (α-SMA) antibody (SMC marker) conjugated to Alexa Fluor 488 (signal shown in green) and DAPI. Macrophage marker antibody were visualized with biotin/streptavidin amplification system using AlexaFluor 594 as a fluorescent reporter (shown in red). Note that each MF marker antibody stained virtually identical cell population in the plaque and cells immunopositive for MSR-A are immunonegative for α-SMA, and *vice versa* (upper left panel). Lu, lumen, TM, tunica media, TA, tunica adventitia, FC. Fibrous cap.

Suppl.Fig.2B

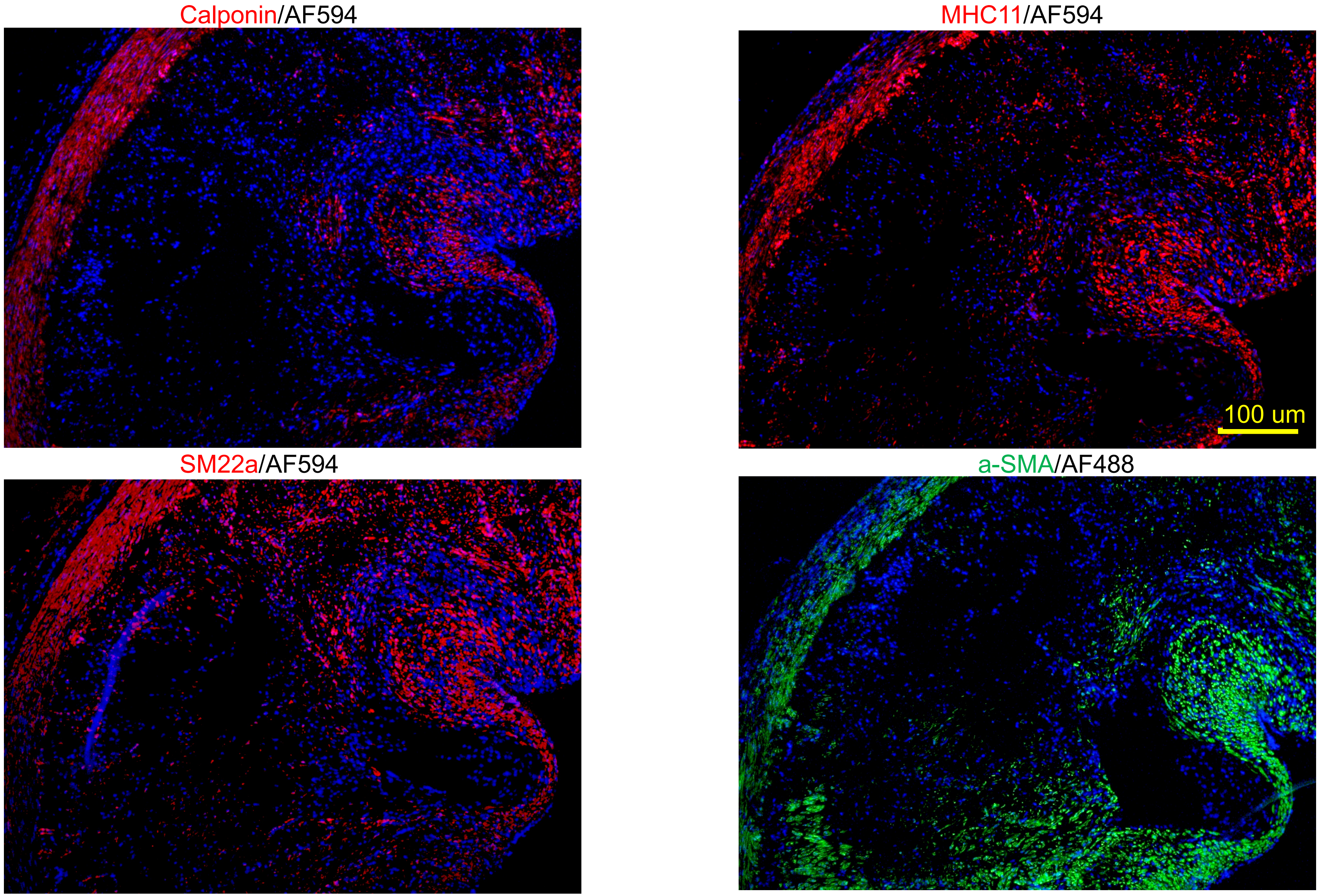

<u>Supplemental Figure 2B</u>. Validation of cell marker antibodies for immunohistochemistry. Serial RCA sections were stained with α-SMA, calponin, smooth muscle protein 22α (SM22α), and myosin heavy chain 11 antibody (all are SMC markers). Each of SMC antibody detected identical cell population in the plaque and vascular media.

Suppl.Fig.2C

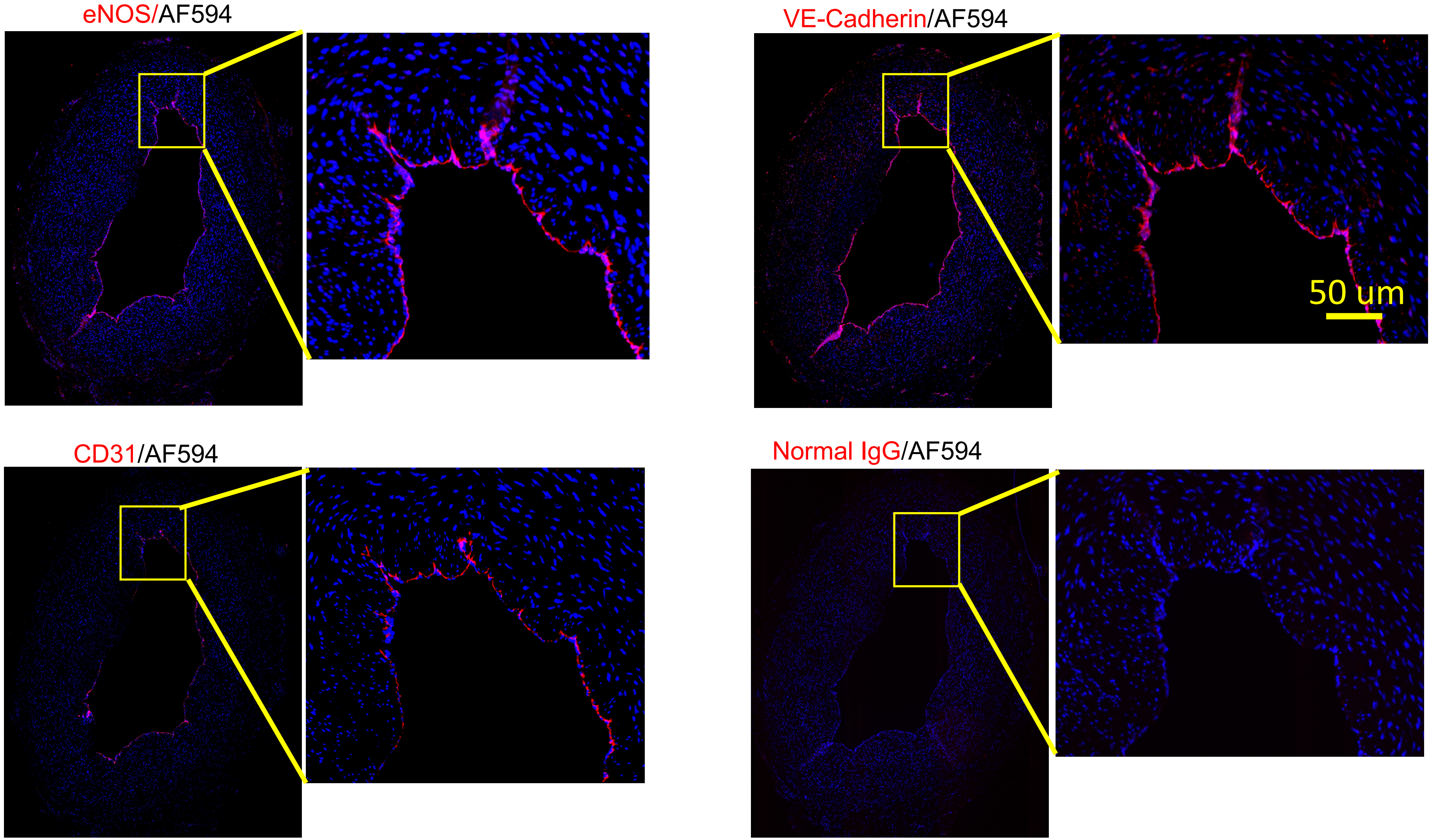

<u>Supplemental Figure 2C</u>. Validation of cell marker antibodies for immunohistochemistry. Serial RCA sections were stained with endothelial nitric oxide synthase (eNOS), CD31, VE-cadherin antibody (all are EC markers). Each of EC antibody detected identical cell subpopulation on the luminal border.

Suppl.Fig.3

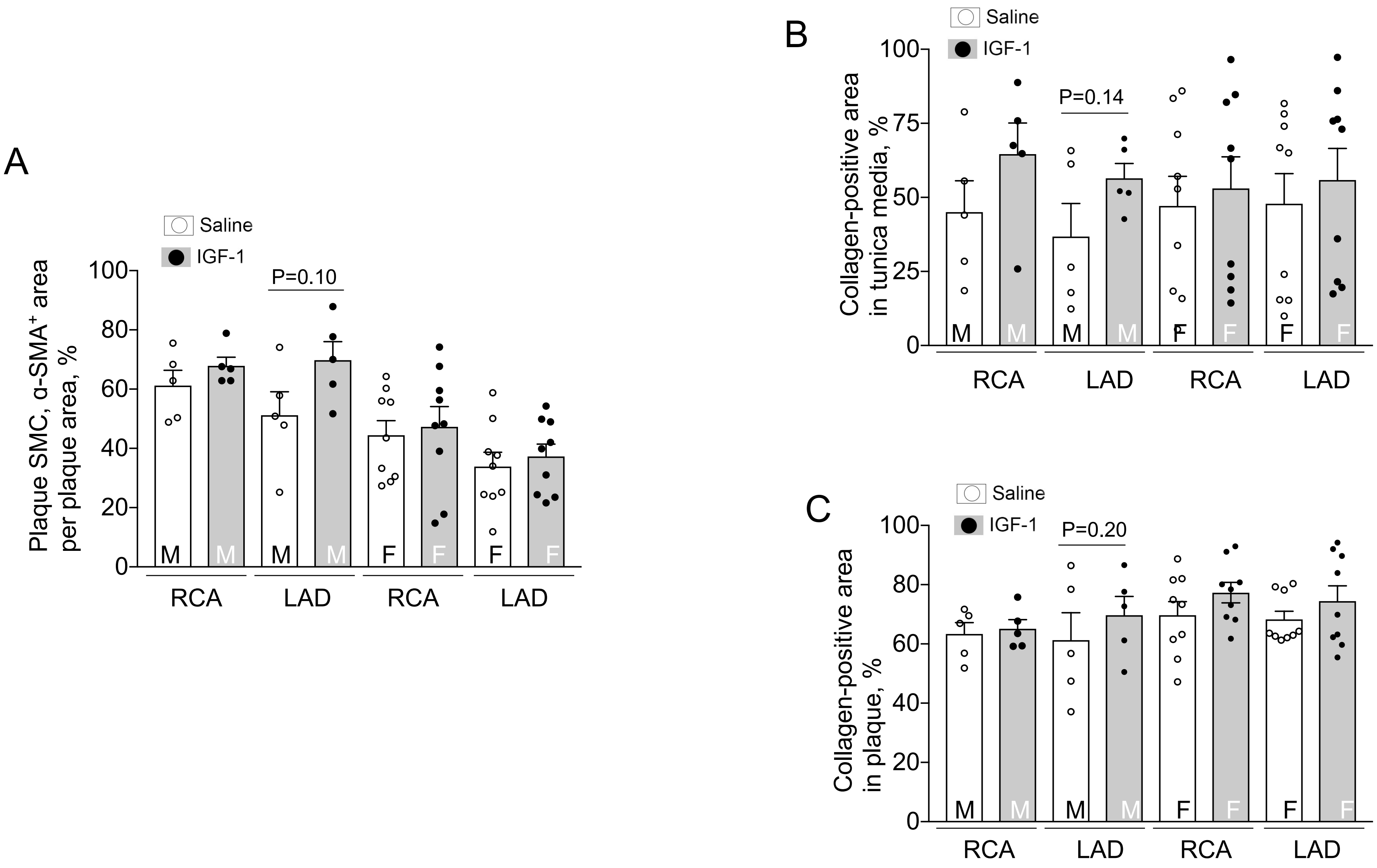

Supplemental Figure 3. IGF-1 effect on plaque SMC and collagen. IGF-1 or saline (control) was injected into FH pigs (N=5, males, N=9, females) and pigs were fed with high-fat diet (HFD). RCA and LAD cross-sections were immunostained with α-smooth muscle actin (α-SMA) antibody (SMC marker) (A) or stained with Gomori's Trichrome stain to visualize collagen in the vascular media and plaque (B, C). Sections were imaged and tunica media and atherosclerotic plaque cross-sectional area were manually outlined. M, males, F, females. P value was calculated based on Student's t-test.

Suppl.Fig.4

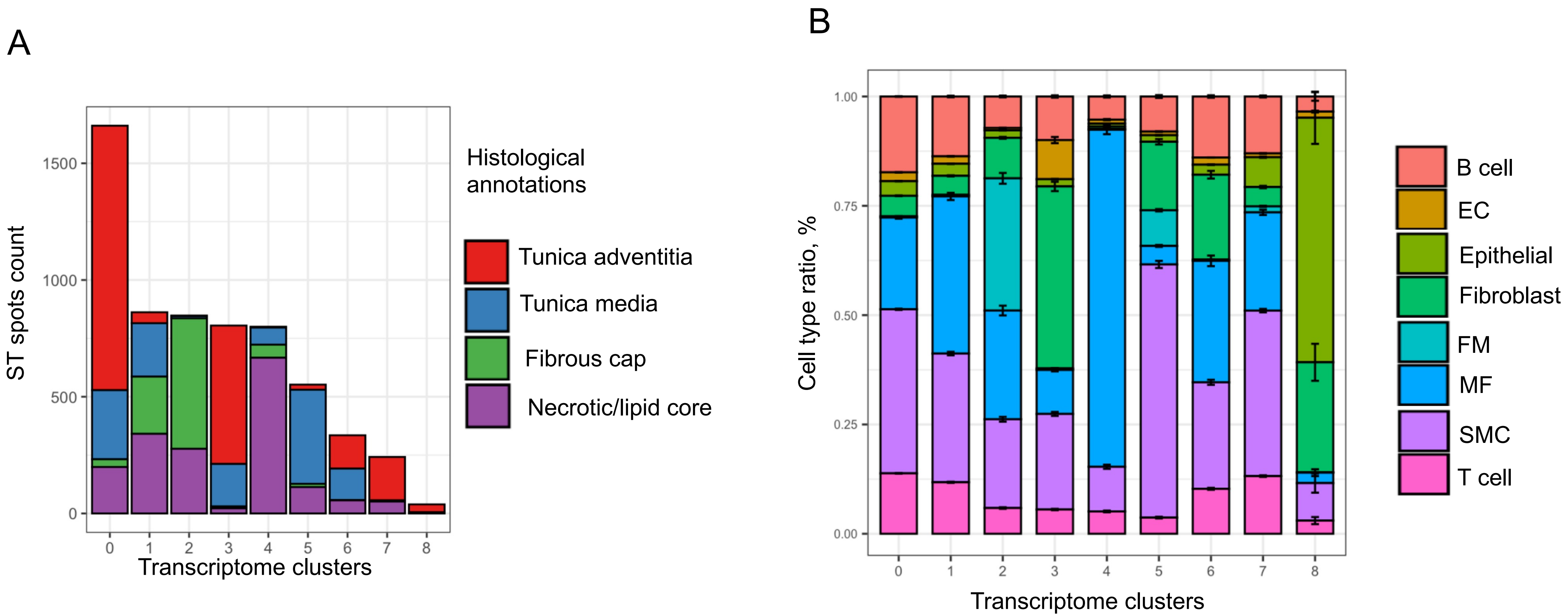

__**Supplemental Figure 4.**__ **Spatial transcriptomics (ST) clustering.** RCA cryosections from IGF-1- and saline-injected FH females (N=2/group) were processed with ST protocol. A, All ST spots in IGF-1 and saline specimens were grouped into 9 clusters (0-8) based on their transcriptome. In parallel, plaque's fibrous cap, necrotic/lipid core, tunica media and tunica adventitia were manually outlined using H&E image and transcriptome clusters and histological annotations were compared side-by-side. ST spots count per each transcriptome cluster is shown. B, cell type ratio (%) was calculated for each ST spot to identify spots enriched by SMC, MF, fibromyocytes (FM), B cells, T cells, epithelial cells, endothelial cells (EC) and fibroblasts.
